# Supplementary material for: Lung Injury in COVID-19 Has Pulmonary Edema as an Important Component and Treatment with Furosemide and Negative Fluid Balance (NEGBAL) Decreases Mortality
Source: J Clin Med. 2023 Feb 15;12(4):1542. doi: 10.3390/jcm12041542 (PMC9966668; doi:10.3390/jcm12041542)
Supplement: Supplementary file 1 [file jcm-12-01542-s001.zip › Santos JLF et al. Supplement 1.pdf]

Appendix: Supplemental Statistic Nº 1

This appendix has been provided by the authors to give readers additional information about their work.

Supplement to: Jose L. Francisco Santos, MD; Patricio Zanardi, MD; Veronica Alo, MD; Vanina Dos Santos, MD; Leonardo Bovone, MD; Marcelo Rodriguez, MD; Federico Magdaleno, MD; Virginia De Langhe, MD; Andrea Villoldo, MD; Romina Martinez Souvielle, MD; Julieta Alconcher, MD; Diego Quiros, MD; Claudio Milicchio, MD and Eduardo Garcia Saiz, MD. **Lung Injury in COVID-19 has Pulmonary Edema as an important component and treatment with Furosemide and Negative Fluid Balance (NEGBAL) decreases mortality.**

Statistical Report
COVID-19 treatment with NEGBAL protocol

Dr. Hernán A. Vivas
Centro Marplatense de Investigaciones Matemáticas (UNMdP - CIC)

Contents

1. **Data source and description**
2. **Statistical model**
3. **Results**

Table S1. AGE summary
Figure S1. Boxplot for AGE
Table S2. Paired differences t–test between AGE CONTROL and AGE NEGBAL
Table S3. WEIGHT summary
Table S4. Paired differences t–test between WEIGHT CONTROL and WEIGHT NEGBAL
Figure S2: Boxplot for WEIGHT
Table S5: SEX frequencies
Table S6: Pearson’s Chi-squared test (Yates’ continuity correction)
Table S7: COVID vaccine, 1st dose frequencies
Table S8: Pearson’s Chi-squared test (Yates’ continuity correction)
Table S9: COVID vaccine, 2nd dose frequencies
Table S10: Pearson’s Chi-squared test (Yates’ continuity correction)
Table S11: OBESITY frequencies
Table S12: Pearson’s Chi-squared test (Yates’ continuity correction)
Table S13: HTA frequencies
Table S14: Pearson’s Chi-squared test (Yates’ continuity correction)
Table S15: DBT frequencies
Table S16: Pearson’s Chi-squared test (Yates’ continuity correction
Table S17: EPOC frequencies
Table S18: Pearson’s Chi-squared test (Yates’ continuity correction
Table S19: CARDIOP frequencies
Table S20: Fisher’s Exact Test
Table S21: HR ADM summary.
Table S22: Paired differences t–test between HR ADM CONTROL and HR ADM NEGBAL
Figure S3: Boxplot for HR ADM.
Table S23: SBP ADM summary
Figure S4: Boxplot for SBP ADM.
Table S24: Paired differences t–test between SBP ADM CONTROL and SBP ADM NEGBAL.
Table S25: DBP ADM summary
Figure S5: Boxplot for DBP ADM.
Table S26: Paired differences t–test between DBP ADM CONTROL and DBP ADM NEGBAL.
Table S27: TEMP ADM summary
Figure S6: Boxplot for TEMP ADM.
Table S28: Paired differences t–test between TEMP ADM CONTROL and TEMP ADM NEGBAL.
Table S29: RR ADM summary.
Table S30: Paired differences t–test between RR ADM CONTROL and RR ADM NEGBAL

Table S31: APACHE ADM summary.
Figure S7: Boxplot for RR ADM.
Figure S8: Boxplot for APACHE ADM.
Table S32: Paired differences t−test between APACHE ADM CONTROL and APACHE ADM NEGBAL.
Table S33: BMI summary.
Figure S9: Boxplot for BMI.
Table S34: Paired differences t−test between BMI CONTROL and BMI NEGBAL.
Table S35: PO2 ADM summary
Table S36: Paired differences t−test between PO2 ADM CONTROL and PO2 ADM NEGBAL
Figure S10: Boxplot for PO2 ADM
Table S37: FiO2 ADM summary.
Figure S11: Boxplot for FiO_2 ADM.
Table S38: Paired differences t−test between FiO2 ADM CONTROL and FiO2 ADM NEGBAL.
Table S39: PaFiO2 ADM summary
Figure S12: Boxplot for PaFiO2 ADM
Table S40: Paired differences t−test between PaFiO2 ADM CONTROL and PaFiO2 ADM NEGBAL.
Table S41: $P_{CO}2$ ADM summary.
Figure S13: Boxplot for $P_{CO}2$ ADM.
Table S42: Paired differences t−test between $P_{CO}2$ ADM CONTROL and $P_{CO}2$ ADM NEGBAL.
Table S43: HCO_3 ADM summary.
Table S44: Paired differences t−test between HCO_3 ADM CONTROL and HCO_3 ADM NEGBAL.
Figure S14: Boxplot for HCO_3 ADM.
Table S45: CREAT ADM summary
Figure S15: Boxplot for CREAT ADM.
Table S46: Paired differences t−test between CREAT ADM CONTROL and CREAT ADM NEGBAL.
Table S47: LYMP ADM summary.
Table S48: LYMP D4 summary.

4. **Data for the whole population**

Table S49: Paired differences t−test between LYMP ADM CONTROL and LYMP D4 CONTROL
Figure S16: Boxplot for LYMP ADM v LYMP D4 for CONTROL group.
Figure S17: Boxplot for LYMP ADM v LYMP D4 for NEGBAL group.
Table S50: Paired differences t−test between LYMP ADM NEGBAL and LYMP D4 NEGBAL.
Table S51: Paired differences t−test between LYMP ADM CONTROL and LYMP ADM NEGBAL
Figure S18: Boxplot for LYMP ADM CONTROL v NEGBAL group.
Figure S19: Boxplot for LYMP D4 CONTROL v NEGBAL group.
Table S52: Paired differences t−test between LYMP D4 CONTROL and LYMP D4 NEGBAL.
Table S53: NAP ADM summary.
Figure S20: Boxplot for NAP ADM.
Table S54: Paired differences t−test between NAP ADM CONTROL and NAP ADM NEGBAL.
Table S55: HTO ADM summary.
Table S56: Paired differences t−test between HTO ADM CONTROL and HTO ADM NEGBAL
Figure S21: Boxplot for HTO ADM.
Table S57: AHT frequencies
Table S58: Pearson’s Chi-squared test (Yates’ continuity correction
Table S59: BNP ADM summary.
Table S60: Paired differences t−test between BNP ADM CONTROL and BNP ADM NEGBAL.
Table S61: TROPO ADM summary.
Figure S22: Boxplot for BNP ADM.
Figure S23: Boxplot for TROPO ADM.
Table S62: Paired differences t−test between TROPO ADM CONTROL and TROPO ADM NEGBAL.
Table S63: TGO ADM summary.
Figure S24: Boxplot for TGO ADM.
Table S64: Paired differences t−test between TGO ADM CONTROL and TGO ADM NEGBAL
Table S65: TGP ADM summary.
Table S66: Paired differences t−test between TGP ADM CONTROL and TGP ADM NEGBAL
Figure S25: Boxplot for TGP ADM.
Table S67: FAL ADM summary.
Figure S26: Boxplot for FAL ADM.
Table S68: Paired differences t−test between FAL ADM CONTROL and FAL ADM NEGBAL
Table S69: BIT ADM summary.

Figure S27: Boxplot for BIT ADM.
Table S70: Paired differences $t-$test between BIT ADM CONTROL and BIT ADM NEGBAL.
Table S71: BID ADM summary.
Figure S28: Boxplot for BID ADM.
Table S72: Paired differences $t-$test between BID ADM CONTROL and BID ADM NEGBAL.
Table S73: Avergage value, maximum, minimum and standard deviation of selected characteristics for the whole population.
Table S74: Percentages of age ranges for the whole population and for CONTROL and NEGBAL groups and $p-$value of the comparison CONTROL v NEGBAL.
Table S75: Proportion of whole population for selected categorical variables.

1 Data source and description

We analyzed data from 116 patients treated for COVID-19 disease. A first group of 58 patients was treated with the standard treatment (CONTROL group) and a second group was treated following the NEGBAL protocol (NEGBAL group) . At this stage, we analyzed data from both populations as to inquire whether there was any statistically significant difference between them, according to the variables under observation.

The variables analyzed were: AGE, WEIGHT, SEX (F or M), vaccination status for 1st and 2nd doses (yes or no), OBESITY (yes or no), DBT (diabetes, yes or no), EPOC (EPOC or tabaquism, yes or no), CARDIOP (cardiopathy, yes or no), HR (heart rate), SBP (systolic blood pressure), DBP (diastolic blood pressure), TEMP (temperature), RR (respiratory rate), APACHE (acute physiology and chronic health evaluation score), BMI (body mass index), PO_2, FiO_2, PaFiO_2, $P_{CO}2$, HCO_3, CREAT (creatinine), LYMP (lymphocytes)[1], NAP, HTO, AHT (yes or no), BNP, TROPO, TGO, TGP, FAL, BIT, BID.

Below, the references NEGBAL and CONTROL refer to which group the variable under consideration belongs and ADM means that the value of the variable was observed at the moment of admission.

2 Statistical model

The comparison between numerical variables was done be via Welch's two samples t test with null hypothesis that the difference of the means equals 0. The comparison between categorical variables was done mostly via a Person's χ^2 test, except for the case of the CARDIOP variable, for which a Fisher's test was used.

3 Results

The results of the statistical analysis are displayed below. For each numerical variable two tables and one plot are provided: the corresponding statistical summary (IQR standing for interquartile range and sd for standard deviation) for the CONTROL and BLANEG groups and the t test for difference of the means and the boxplot for both samples. For the categorical variables, a contingency table is provided, and on its caption the result of the χ^2 test (Fisher in the case of CARDIOP).

[1]Data for one patient presenting extreme values was eliminated from this analysis by clinical consideration (leukemoid reaction).

Table S1: AGE summary

AGE		
	CONTROL GROUP (N = 58)	NEGBAL GROUP (N = 58)
minimum	27.00	22.00
median (IQR)	62.5 (49.25, 68.75)	57.5.00 (48.00, 73.75)
mean (sd)	59.45 ± 12.04	59.86 ± 15.14
maximum	78.00	91.00

Figure S1: Boxplot for AGE

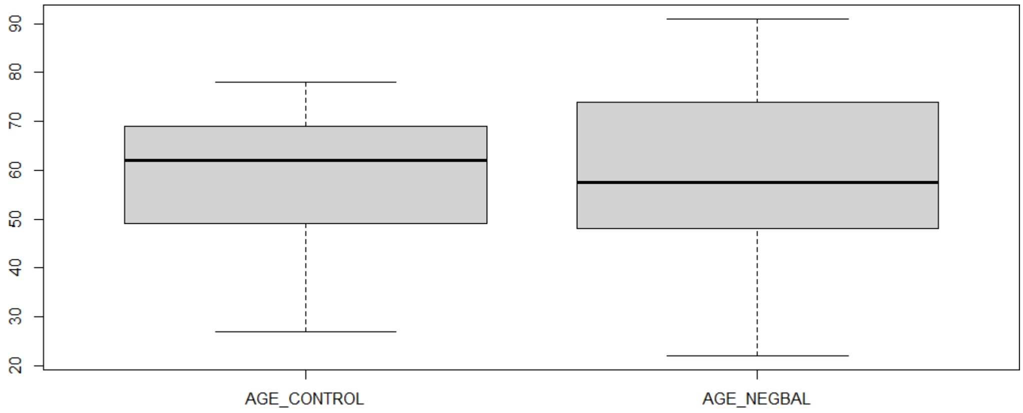

Table S2: Paired differences t−test between AGE CONTROL and AGE NEGBAL.

**AGE CONTROL v AGE BALNEG**				
t	DF	*p*−value	95% confidence interval	sample estimates (respectively)
-0.16294	108.49	0.8709	[-5.447341, 4.619755]	59.44828 59.86207

Table S3: WEIGHT summary

WEIGHT		
	CONTROL GROUP (N = 58)	NEGBAL GROUP (N = 58)
minimum	50.00	50.00
median (IQR)	85 (100, 74.5)	84.5 (74.25, 99.00)
mean (sd)	87.33 ± 19.4	87.2 ± 17.93
maximum	130.00	136.00

Table S4: Paired differences t−test between WEIGHT CONTROL and WEIGHT NEGBAL

**WEIGHT CONTROL v WEIGHT NEGBAL**				
t	DF	*p*−value	95% confidence interval	sample estimates (respectively)
0.036534	113.3	0.9709	[-6.745129, 6.998578]	87.32759 87.20086

Figure S2: Boxplot for WEIGHT

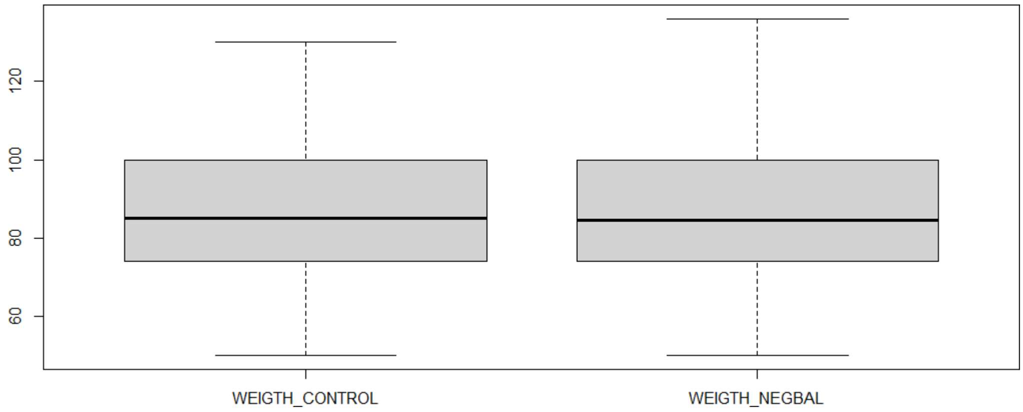

Table S5: SEX frequencies

	F	M
CONTROL	18	40
NEGBAL	22	36

Table S6: Pearson's Chi-squared test (Yates' continuity correction): χ^2= 0.34342, df = 1, p−value = 0.5579

Table S7: COVID vaccine, 1st dose frequencies

	Yes	No
CONTROL	18	40
NEGBAL	34	24

Table S8: Pearson's Chi-squared test (Yates' continuity correction): χ^2= 7.8425, df = 1, p−value = 0.005103

Table S9: COVID vaccine, 2nd dose frequencies

	Yes	No
CONTROL	5	53
NEGBAL	22	36

Table S10: Pearson's Chi-squared test (Yates' continuity correction): χ^2= 12.358, df = 1, p−value = 0.0004391

Table S11: OBESITY frequencies

	Yes	No
CONTROL	30	28
NEGBAL	29	29

Table S12: Pearson's Chi-squared test (Yates' continuity correction): χ^2= 0, df = 1, $p-$value = 1

Table S13: HTA frequencies

	Yes	No
CONTROL	23	35
NEGBAL	24	34

Table S14: Pearson's Chi-squared test (Yates' continuity correction): χ^2= 0, df = 1, $p-$value = 1

Table S15: DBT frequencies

	Yes	No
CONTROL	17	41
NEGBAL	13	45

Table S16: Pearson's Chi-squared test (Yates' continuity correction): χ^2= 0.40465, df = 1, $p-$value = 0.5247

Table S17: EPOC frequencies

	Yes	No
CONTROL	10	48
NEGBAL	10	47

Table S18: Pearson's Chi-squared test (Yates' continuity correction): χ^2= 1.3287x10^{-31}, df = 1, p value = 0.5247

Table S19: CARDIOP frequencies

	Yes	No
CONTROL	4	54
NEGBAL	0	58

Table S20: Fisher's Exact Test: $p-$value = 0.1185, 95% percent confidence interval: [0, 1.482778]

Table S21: HR ADM summary.

HR		
	CONTROL GROUP (N = 58)	NEGBAL GROUP (N = 58)
minimum	56.00	53.00
median (IQR)	83.5 (75.00, 96.00)	81.00 (71.25, 94.75)
mean (sd)	84.55 ± 14.9	82.5 ± 15.19
maximum	120.00	118.00

Table S22: Paired differences t−test between HR ADM CONTROL and HR ADM NEGBAL

HR ADM CONTROL v HR ADM NEGBAL				
t	DF	*p*−value	95% confidence interval	sample estimates (respectively)
0.73446	113.96	0.4642	[-3.482200, 7.585648]	84.55172 82.50000

Figure S3: Boxplot for HR ADM.

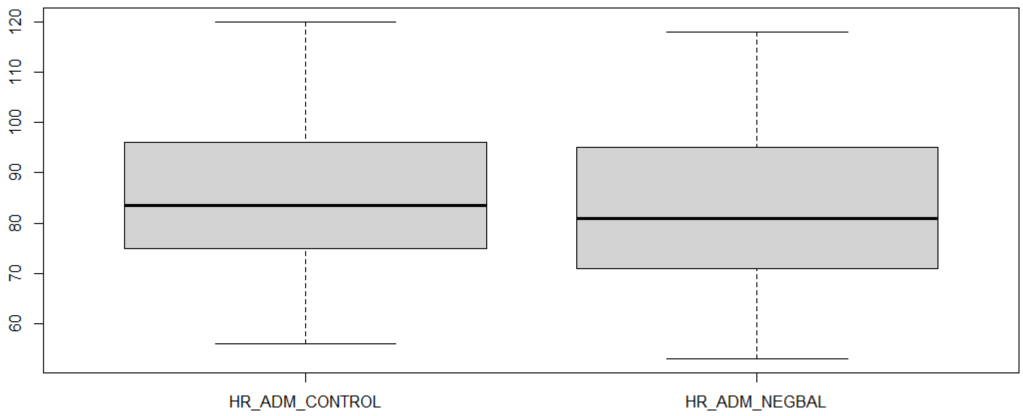

Table S23: SBP ADM summary

SBP		
	CONTROL GROUP (N = 58)	NEGBAL GROUP (N = 58)
minimum	93.00	95.00
median (IQR)	121.00 (115.00, 134.8)	126.00 (120.00, 138.00)
mean (sd)	127.2 ± 20.77	128.3 ± 17.75
maximum	223.00	173.00

Figure S4: Boxplot for SBP ADM.

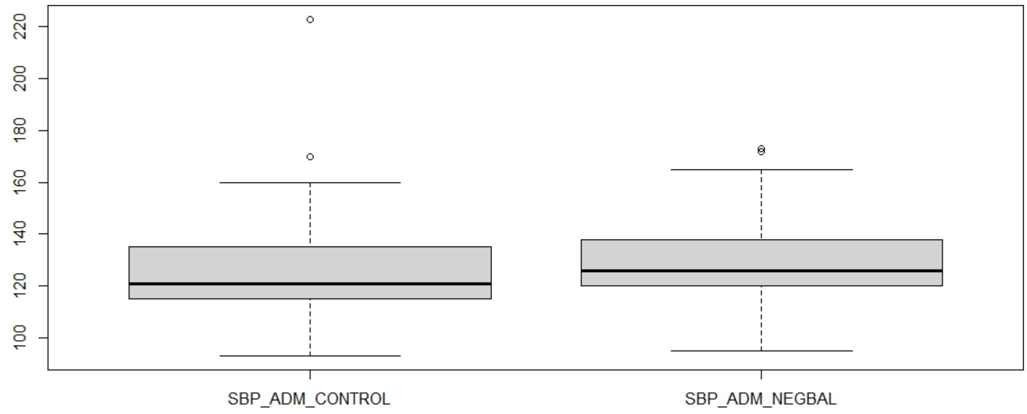

Table S24: Paired differences t−test between SBP ADM CONTROL and SBP ADM NEGBAL.

SBP ADM CONTROL v SBP ADM NEGBAL				
t	DF	p−value	95% confidence interval	sample estimates (respectively)
-0.30757	111.3	0.759	[-3.482200, 7.585648]	127.2241 128.3276

Table S25: DBP ADM summary

DBP		
	CONTROL GROUP (N = 58)	NEGBAL GROUP (N = 58)
minimum	44.00	58.00
median (IQR)	76.00 (70.00, 84.00)	79.00 (70.00, 85.75)
mean (sd)	76.79 ± 12.47	78.14 ± 12.4
maximum	115.00	105.00

Figure S5: Boxplot for DBP ADM.

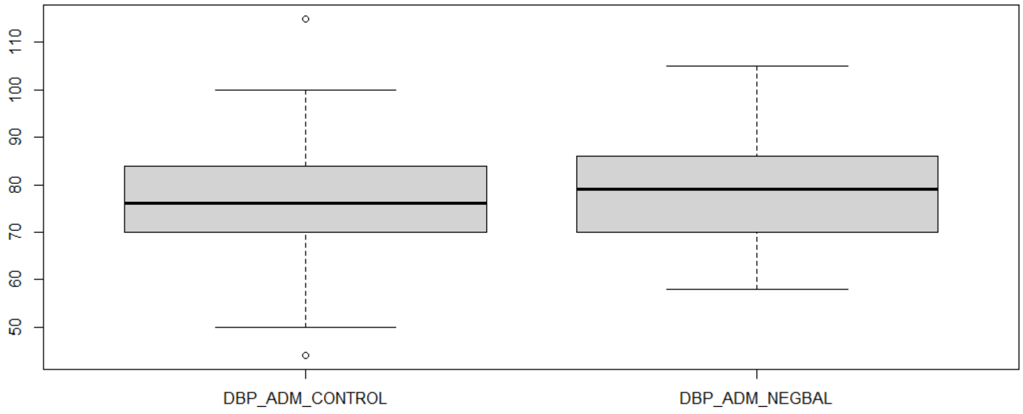

Table S26: Paired differences t−test between DBP ADM CONTROL and DBP ADM NEGBAL.

DBP ADM CONTROL v DBP ADM NEGBAL				
t	DF	p−value	95% confidence interval	sample estimates (respectively)
-0.58225	114	0.5615	[-5.920307, 3.230652]	76.79310 78.13793

Table S27: TEMP ADM summary

	CONTROL GROUP (N = 58)	NEGBAL GROUP (N = 58)
	**TEMP**	
minimum	35.00	35.00
median (IQR)	36.4 (36.00, 37.38)	36.25 (36.00, 36.9)
mean (sd)	36.63 ± 0.88	36.43 ± 0.84
maximum	38.2	39.00

Figure S6: Boxplot for TEMP.ADM.

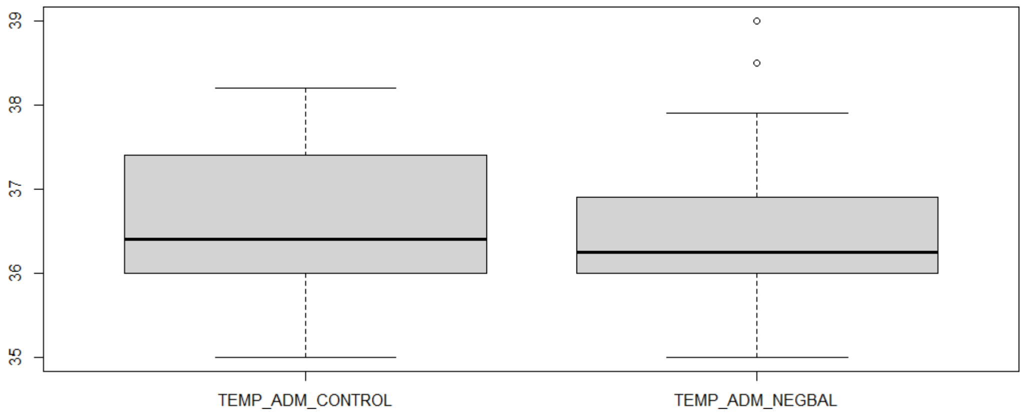

Table S28: Paired differences $t-$test between TEMP ADM CONTROL and TEMP ADM NEGBAL.

TEMP ADM CONTROL v TEMP ADM NEGBAL

t	DF	*p*−value	95% confidence interval	sample estimates (respectively)
1.2882	113.78	0.2003	[-0.1103479, 0.5206927]	36.63276 36.42759

Table S29: RR ADM summary.

	CONTROL GROUP (N = 58)	NEGBAL GROUP (N = 58)
	**RR**	
minimum	16.00	13.00
median (IQR)	25.00 (22.00, 28.00)	25.00 (22.00, 26.00)
mean (sd)	25.34 ± 4.61	24.47 ± 4.56
maximum	40.00	35.00

Table S30: Paired differences $t-$test between RR ADM CONTROL and RR ADM NEGBAL

RR ADM CONTROL v RR ADM NEGBAL

t	DF	*p*−value	95% confidence interval	sample estimates (respectively)
1.033	113.99	0.3038	[-0.8069831, 2.5656038]	25.34483 24.46552

Table S31: APACHE ADM summary

APACHE		
	CONTROL GROUP (N = 58)	NEGBAL GROUP (N = 58)
minimum	3.00	3.00
median (IQR)	8.00 (5.00, 9.00)	8.00 (6.00, 10.75)
mean (sd)	7.6 ± 3.04	8.43 ± 3.44
maximum	15.00	21.00

Figure S7: Boxplot for RR ADM.

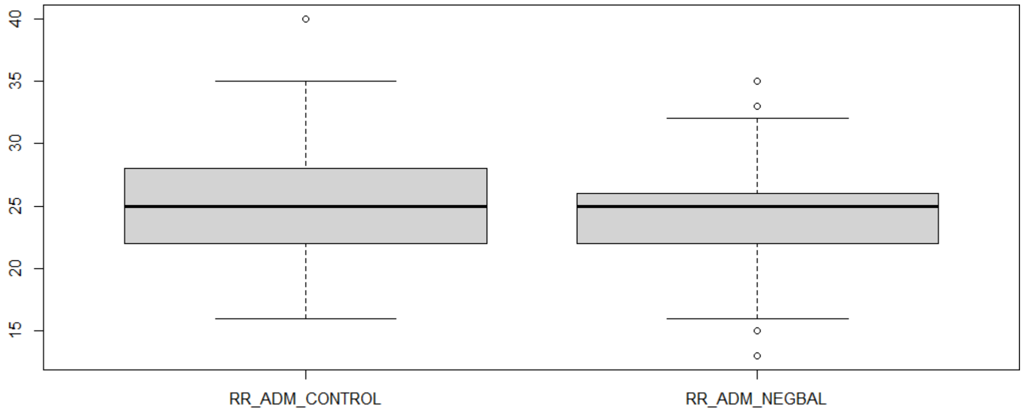

Figure S8: Boxplot for APACHE ADM.

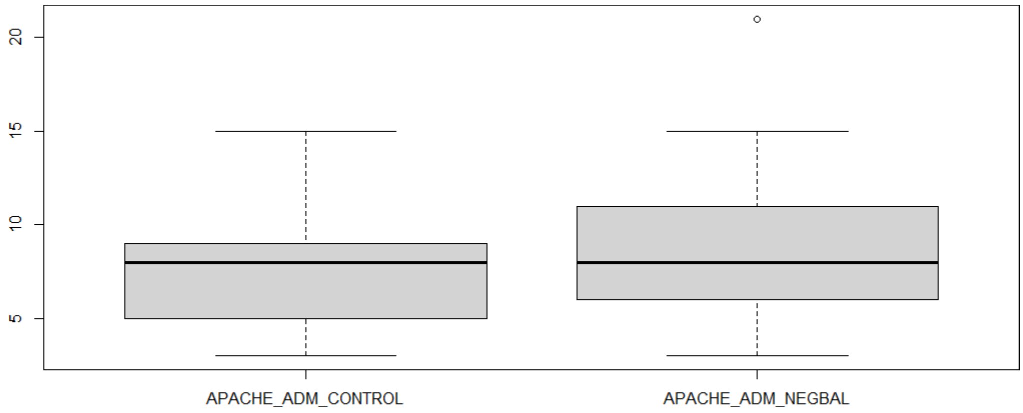

Table S32: Paired differences $t-$test between APACHE ADM CONTROL and APACHE ADM NEGBAL

APACHE ADM CONTROL v APACHE ADM NEGBAL				
t	DF	$p-$value	95% confidence interval	sample estimates (respectively)
-1.033	112.3	0.173	[-2.0233185, 0.3681461]	7.603448 8.431034

Table S33: BMI summary.

**BMI**		
	CONTROL GROUP (N = 58)	NEGBAL GROUP (N = 58)
minimum	19.00	22.00
median (IQR)	29.5 (26, 33.75)	30.00 (26.00, 33.00)
mean (sd)	30.25 ± 5.46	29.93 ± 4.89
maximum	42.00	41.00

Figure S9: Boxplot for BMI.

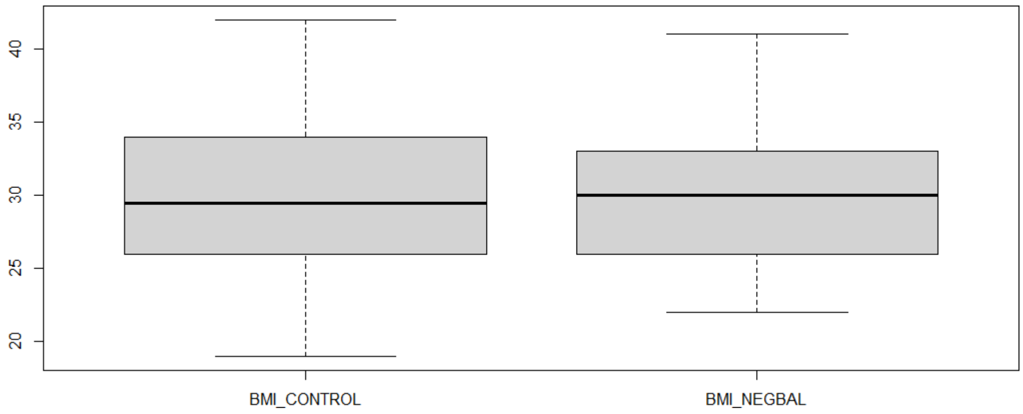

Table S34: Paired differences t−test between BMI CONTROL and BMI NEGBAL.

**BMI CONTROL v BMI NEGBAL**				
t	DF	p−value	95% confidence interval	sample estimates (respectively)
0.3318	112.57	0.7407	[-1.585684, 2.223615]	30.25000 29.93103

Table S35: PO_2 ADM summary

**PO_2 ADM**		
	CONTROL GROUP (N = 58)	NEGBAL GROUP (N = 58)
minimum	43.00	49.00
median (IQR)	68.45 (60.33, 79.22)	69.5 (61.85, 81.75)
mean (sd)	73.99 ± 21.55	72.8 ± 15.97
maximum	149.6	135.00

Table S36: Paired differences t−test between PO_2 ADM CONTROL and PO_2 ADM NEGBAL

**PO_2 ADM CONTROL v PO_2 ADM NEGBAL**				
t	DF	p−value	95% confidence interval	sample estimates (respectively)
0.33829	105.1	0.7358	[-5.791514, 8.174272]	73.98793 72.79655

Figure S10: Boxplot for PO_2 ADM

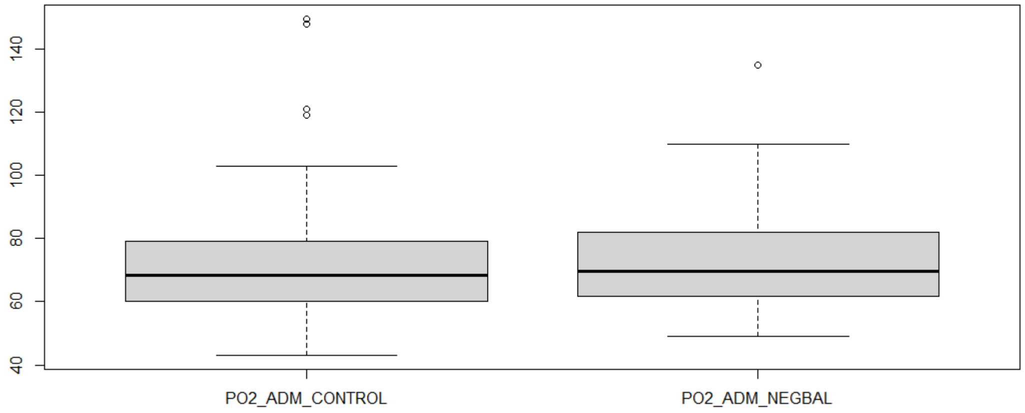

Table S37: FiO_2 ADM summary.

**FiO_2 ADM**		
	CONTROL GROUP (N = 58)	NEGBAL GROUP (N = 58)
minimum	0.24	0.28
median (IQR)	0.45 (0.32, 0.70)	0.40 (0.32, 0.70)
mean (sd)	0.53 ± 0.23	0.53 ± 0.26
maximum	1.00	1.00

Figure S11: Boxplot for FiO_2 ADM.

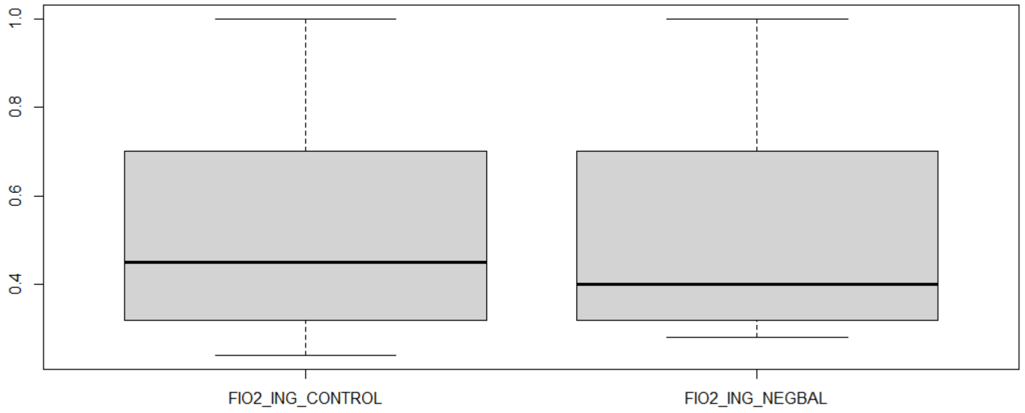

Table S38: Paired differences t−test between FiO_2 ADM CONTROL and FiO_2 ADM NEGBAL.

**FiO_2 ADM CONTROL v FiO_2 ADM NEGBAL**				
t	DF	p−value	95% confidence interval	sample estimates (respectively)
-0.030253	112.41	0.9759	[-0.09171093, 0.08895231]	0.5324138 0.5337931

Table S39: PaFiO_2 ADM summary

PaFiO_2 ADM

	CONTROL GROUP (N = 58)	NEGBAL GROUP (N = 58)
minimum	57.00	54.00
median (IQR)	152.50 (97.85, 228.37)	167.20 (102.30, 221.20)
mean (sd)	164.81 ± 73.64	163.6 ± 67.26
maximum	297.92	281.6

Figure S12: Boxplot for PaFiO_2 ADM.

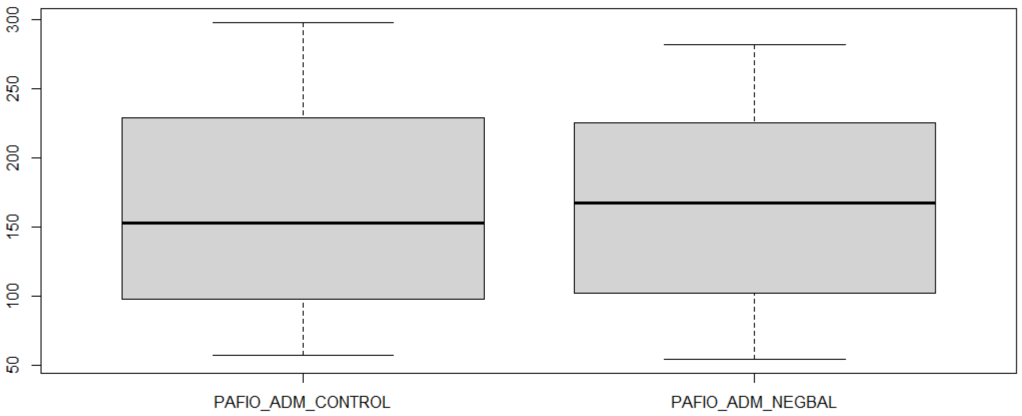

Table S40: Paired differences t−test between PaFiO_2 ADM CONTROL and PaFiO_2 ADM NEGBAL.

PaFiO_2 ADM CONTROL v PaFiO_2 ADM NEGBAL

t	DF	p−value	95% confidence interval	sample estimates (respectively)
0.095144	113.07	0.9244	[-24.69880, 27.19075]	164.8064 163.5604

Table S41: $P_{CO}2$ ADM summary.

$P_{CO}2$ ADM

	CONTROL GROUP (N = 58)	NEGBAL GROUP (N = 58)
minimum	17.00	20.40
median (IQR)	33.50 (30.25, 35.00)	33.00 (29.85,35.00)
mean (sd)	33.75 ± 5.40	32.81 ± 4.90
maximum	48.00	50.00

Figure S13: Boxplot for $P_{CO}2$ -ADM.

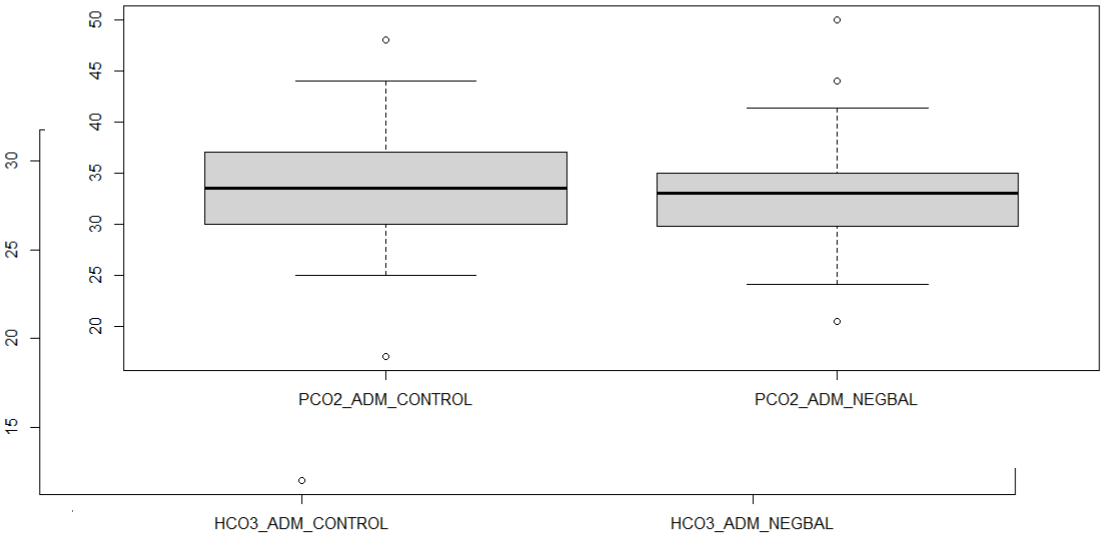

Table S42: Paired differences $t-$test between $P_{CO}2$ ADM CONTROL and $P_{CO}2$ ADM NEGBAL.

$P_{CO}2$ **ADM CONTROL v** $P_{CO}2$ **ADM NEGBAL**

t	DF	$p-$value	95% confidence interval	sample estimates (respectively)
0.98508	112.92	0.3267	[-0.9536765, 2.8398834]	33.74828 32.80517

Table S43: HCO_3 ADM summary.

HCO_3 **ADM**

	CONTROL GROUP (N = 58)	NEGBAL GROUP (N = 58)
minimum	12.00	15.10
median (IQR)	21.50 (20.00, 23.00)	22.00 (20.02,23.80)
mean (sd)	21.64 ± 3.01	23.14 ± 3.12
maximum	31.00	30.00

Table S44: Paired differences $t-$test between HCO_3 ADM CONTROL and HCO_3 ADM NEGBAL.

HCO_3 **ADM CONTROL v** HCO_3 **ADM NEGBAL**

t	DF	$p-$value	95% confidence interval	sample estimates (respectively)
-0.86919	113.86	0.3866	[-1.6226207, 0.6329655]	21.64483 22.13966

Figure S14: Boxplot for HCO_3-ADM.

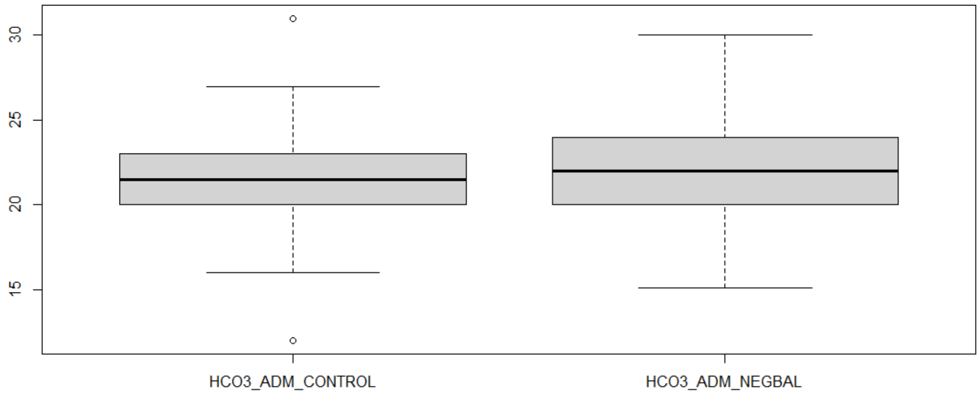

Table S45: CREAT ADM summary

CREAT ADM		
	CONTROL GROUP (N = 58)	NEGBAL GROUP (N = 58)
minimum	5.40	5.20
median (IQR)	8.95 (8.02, 10.25)	8.25 (7.02,10.20)
mean (sd)	9.41 ± 2.38	8.75 ± 2.01
maximum	15.30	13.10

Figure S15: Boxplot for CREAT ADM.

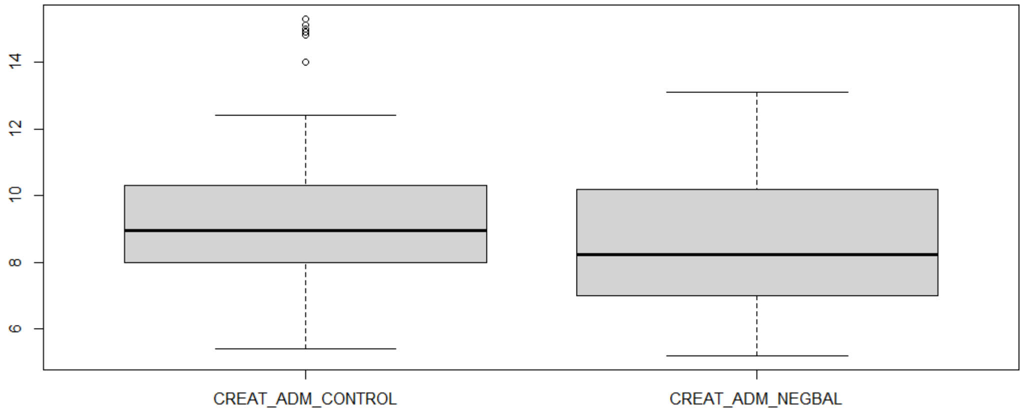

Table S46: Paired differences t−test between CREAT ADM CONTROL and CREAT ADM NEGBAL.

CREAT ADM CONTROL v CREAT ADM NEGBAL				
t	DF	p−value	95% confidence interval	sample estimates (respectively)
1.6081	110.81	0.1107	[-0.1529826, 1.4702240]	9.408621 8.750000

Table S47: LYMP ADM summary.

LYMP ADM		
	CONTROL GROUP (N = 58)	NEGBAL GROUP (N = 57)
minimum	174.0	122.0
median (IQR)	841.5 (570.2, 1150.8)	885.0 (607.0,1200.0)
mean (sd)	963.8 ± 773.0662	955.5 ± 540.31
maximum	5773.0	3700.0

Table S48: LYMP D4 summary.

LYMP D4		
	CONTROL GROUP (N = 56)	NEGBAL GROUP (N = 53)
minimum	183.0	153.0
median (IQR)	642.5 (434.0, 795.0)	946.0 (608.0,1300.0)
mean (sd)	697.1 ± 402.4488	1056.00 ± 735.1
maximum	2059.0	4964

4 Data for the whole population

Table S49: Paired differences t−test between LYMP ADM CONTROL and LYMP D4 CONTROL

LYMP ADM CONTROL v LYMP D4 CONTROL

t	DF	p−value	95% confidence interval	sample estimates (respectively)
2.3225	86.432	0.02256	[38.44399, 495.13852]	963.8448 697.0536

Figure S16: Boxplot for LYMP ADM v LYMP D4 for CONTROL group.

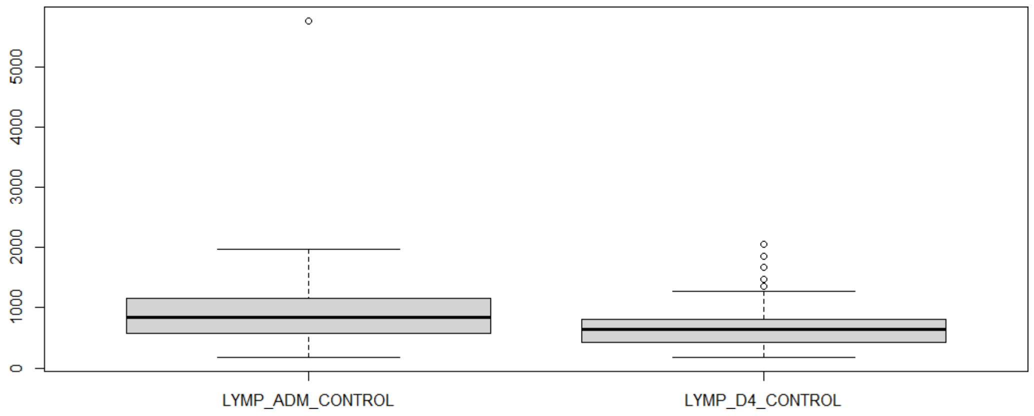

Figure S17: Boxplot for LYMP ADM v LYMP D4 for NEGBAL group.

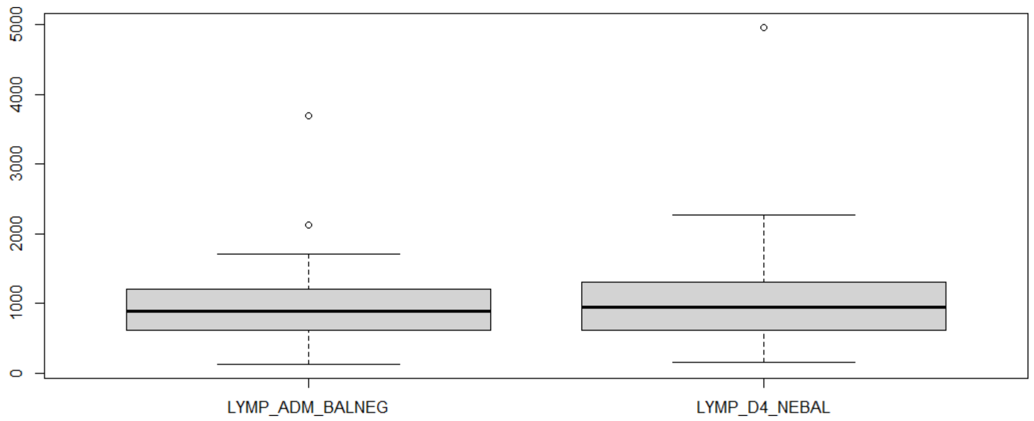

Table S50: Paired differences t−test between LYMP ADM NEGBAL and LYMP D4 NEGBAL.

**LYMP ADM NEGBAL v LYMP D4 NEGBAL**				
t	DF	p−value	95% confidence interval	sample estimates (respectively)
-0.80878	95.085	0.4207	[-345.7983, 145.6030]	955.5439 1055.6415

Table S51: Paired differences t−test between LYMP ADM CONTROL and LYMP ADM NEGBAL

**LYMP ADM CONTROL v LYMP ADM NEGBAL**				
t	DF	p−value	95% confidence interval	sample estimates (respectively)
0.066835	102.08	0.9468	[-238.0483, 254.6502]	963.8448 955.5439

Figure S18: Boxplot for LYMP ADM CONTROL v NEGBAL group.

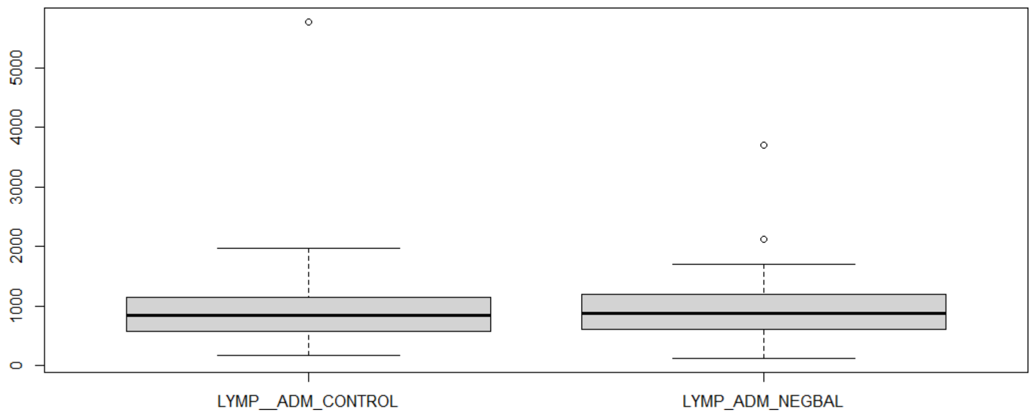

Figure S19: Boxplot for LYMP D4 CONTROL v NEGBAL group.

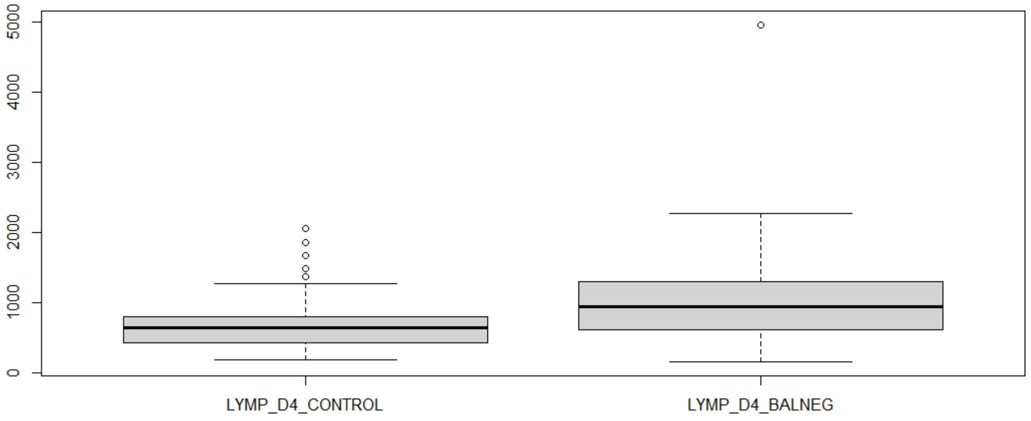

Table S52: Paired differences t−test between LYMP D4 CONTROL and LYMP D4 NEGBAL.

LYMP D4 CONTROL v LYMP D4 NEGBAL

t	DF	p−value	95% confidence interval	sample estimates (respectively)
-3.1344	79.628	0.002411	[-586.2735, -130.9024]	697.0536 1055.6415

Table S53: NAP ADM summary.

NAP ADM

	CONTROL GROUP (N = 58)	NEGBAL GROUP (N = 58)
minimum	128.00	126.00
median (IQR)	136.00 (134.20, 139.00)	136.00 (134.00,137.80)
mean (sd)	136.30 ± 135.50	3.30 ± 2.01
maximum	144.00	142.00

Figure S20: Boxplot for NAP ADM.

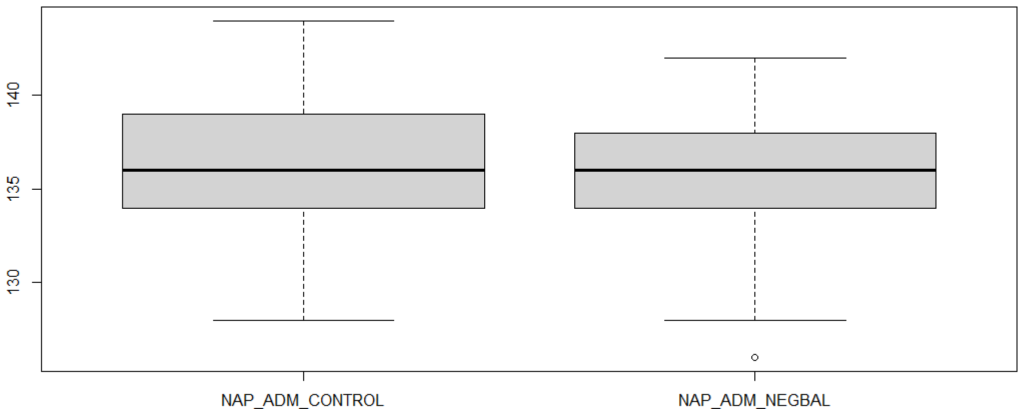

Table S54: Paired differences t−test between NAP ADM CONTROL and NAP ADM NEGBAL.

**NAP ADM CONTROL v NAP ADM NEGBAL**				
t	DF	p−value	95% confidence interval	sample estimates (respectively)
1.3427	113.69	0.182	[-0.4016602, 2.0913153]	136.3103 135.4655

Table S55: HTO ADM summary.

**HTO ADM**		
	CONTROL GROUP (N = 58)	NEGBAL GROUP (N = 58)
minimum	26.00	25.00
median (IQR)	40.80 (38.20,43.90)	40.00 (36.92,42.30)
mean (sd)	40.38 ± 5.22	39.20 ± 4.54
maximum	52.00	45.00

Table S56: Paired differences t−test between HTO ADM CONTROL and HTO ADM NEGBAL

**HTO ADM CONTROL v HTO ADM NEGBAL**				
t	DF	p−value	95% confidence interval	sample estimates (respectively)
1.294	111.84	0.1983	[-0.6247027, 2.9764269]	40.37931 39.20345

Figure S21: Boxplot for HTO ADM.

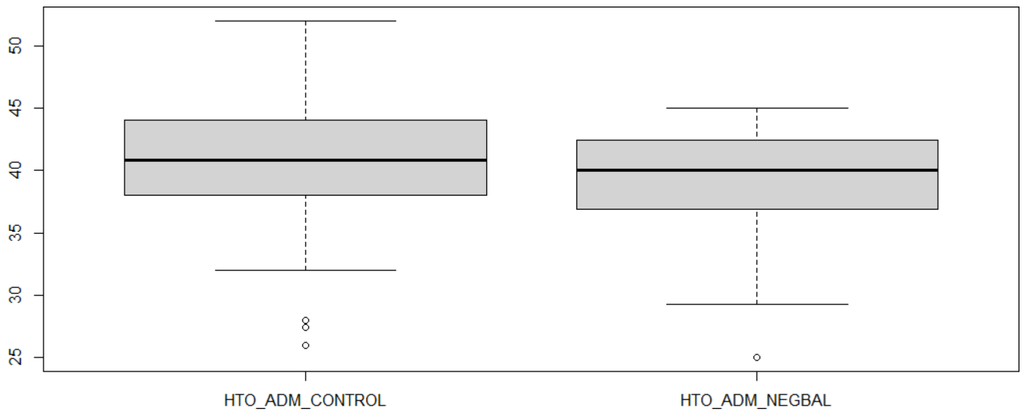

Table S57: AHT frequencies

	Yes	No
CONTROL	24	34
NEGBAL	23	35

Table S58: Pearson's Chi-squared test (Yates' continuity correction): χ^2= 0, df = 1, p−value = 1

Table S59: BNP ADM summary.

BNP ADM

	CONTROL GROUP (N = 31)	NEGBAL GROUP (N = 45)
minimum	10	5
median (IQR)	218 (90,427)	80 (35,280)
mean (sd)	277 ± 246.19	188.80 ± 222.86
maximum	850	873

Table S60: Paired differences t−test between BNP ADM CONTROL and BNP ADM NEGBAL

BNP ADM CONTROL v BNP ADM NEGBAL

t	DF	p−value	95% confidence interval	sample estimates (respectively)
1.5941	60.325	0.1161	[-22.45233, 198.78136]	276.9645 188.8000

Table S61: TROPO ADM summary.

TROPO ADM

	CONTROL GROUP (N = 42)	NEGBAL GROUP (N = 45)
minimum	0.43	3.00
median (IQR)	9.00 (6.00,11.75)	7.00 (5.00,12.00)
mean (sd)	10.84 ± 8.53	9.99 ± 8.25
maximum	41.00	41.00

Figure S22: Boxplot for BNP ADM.

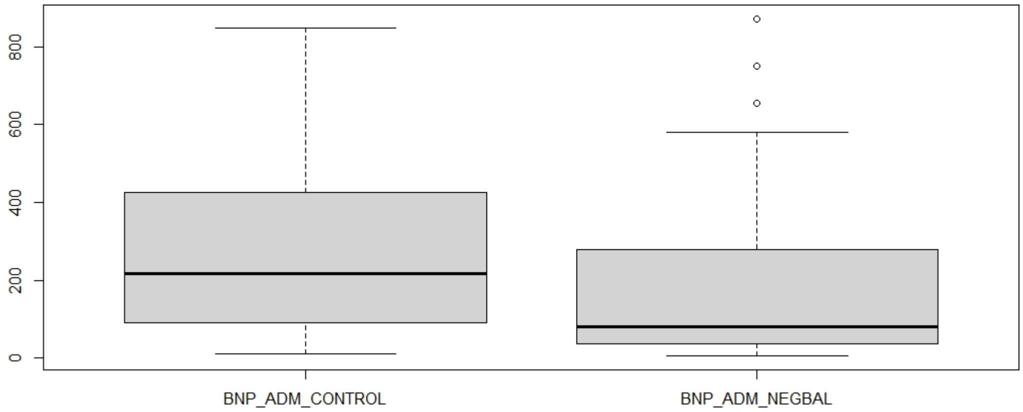

Figure S23: Boxplot for TROPO.ADM.

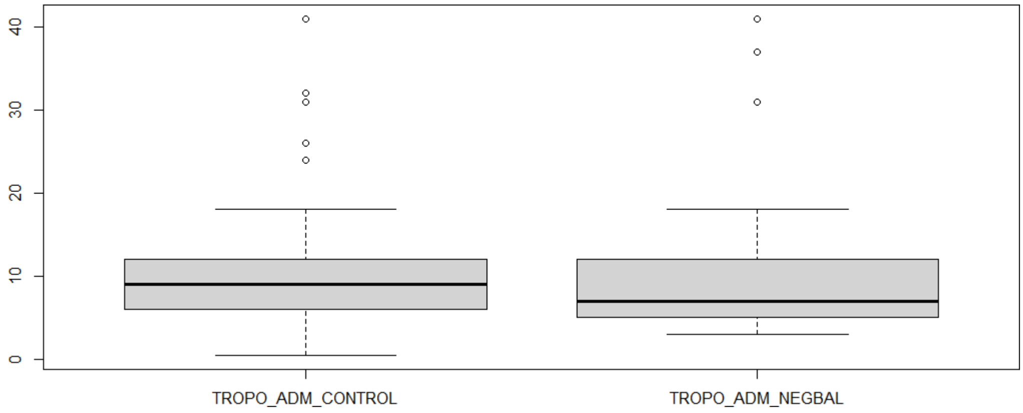

Table S62: Paired differences $t-$test between TROPO ADM CONTROL and TROPO ADM NEGBAL.

TROPO ADM CONTROL v TROPO ADM NEGBAL

t	DF	$p-$value	95% confidence interval	sample estimates (respectively)
0.47087	84.107	0.639	[-2.734726, 4.431647]	10.843571 9.995111

Table S63: TGO ADM summary.

TGO ADM

	CONTROL GROUP (N = 49)	NEGBAL GROUP (N = 45)
minimum	19.00	12.00
median (IQR)	43.00 (29.00,66.00)	34.00 (25.00,55.00)
mean (sd)	53.84 ± 38.85	45.15 ± 36.39
maximum	211.00	232.00

Figure S24: Boxplot for TGO.ADM.

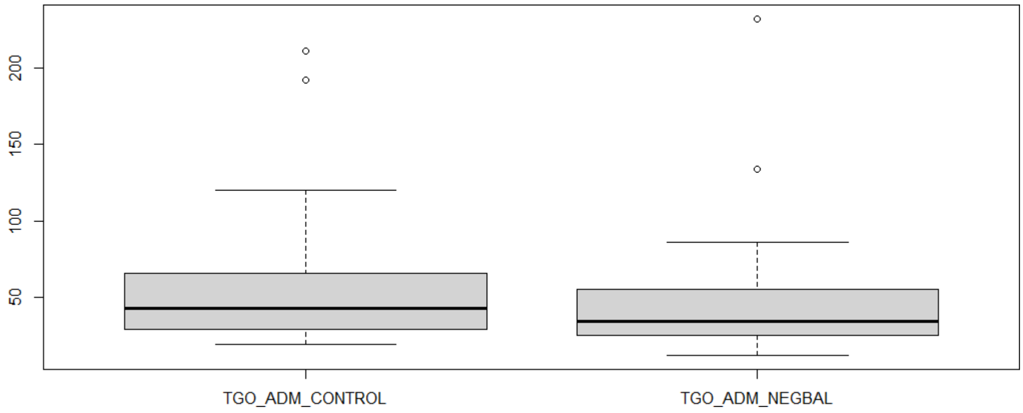

Table S64: Paired differences $t-$test between TGO ADM CONTROL and TGO ADM NEGBAL.

TGO ADM CONTROL v TGO ADM NEGBAL				
t	DF	$p-$value	95% confidence interval	sample estimates (respectively)
1.1312	93.949	0.2608	[-6.560989, 23.936586]	53.83673 45.14894

Table S65: TGP ADM summary.

TGP ADM		
	CONTROL GROUP (N = 49)	NEGBAL GROUP (N = 47)
minimum	10.00	11.00
median (IQR)	45.00 (26.00,78.00)	32.00 (22.00,57.50)
mean (sd)	60.69 ± 64.60	53.38 ± 71.96
maximum	449.00	460.00

Table S66: Paired differences $t-$test between TGP ADM CONTROL and TGP ADM NEGBAL

TGP ADM CONTROL v TGP ADM NEGBAL				
t	DF	$p-$value	95% confidence interval	sample estimates (respectively)
0.54208	91.948	0.5891	[-20.18191, 35.33432]	60.95918 53.38298

Figure S25: Boxplot for TGP ADM.

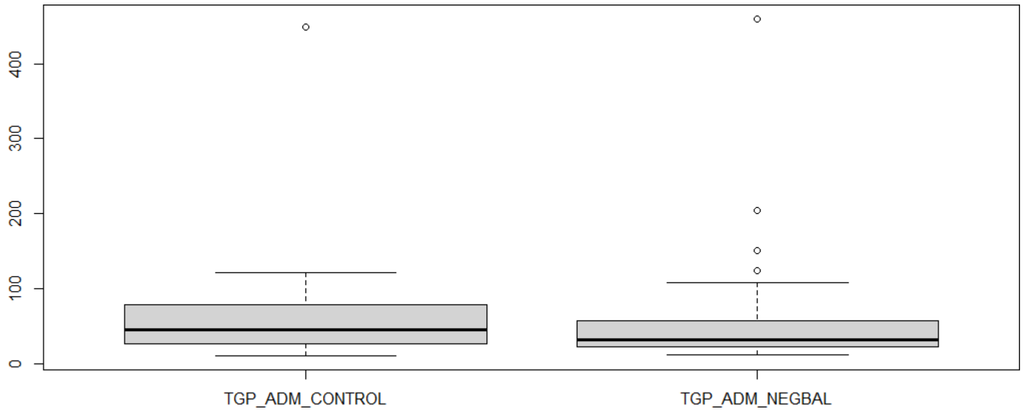

Table S67: FAL ADM summary.

FAL ADM		
	CONTROL GROUP (N = 49)	NEGBAL GROUP (N = 46)
minimum	77.00	60.00
median (IQR)	162.00 (121.00,204.00)	164.00 (60.00,219.20)
mean (sd)	205.20 ± 196.18	185.40 ± 81.31
maximum	1342.00	438.00

Figure S26: Boxplot for FAL ADM.

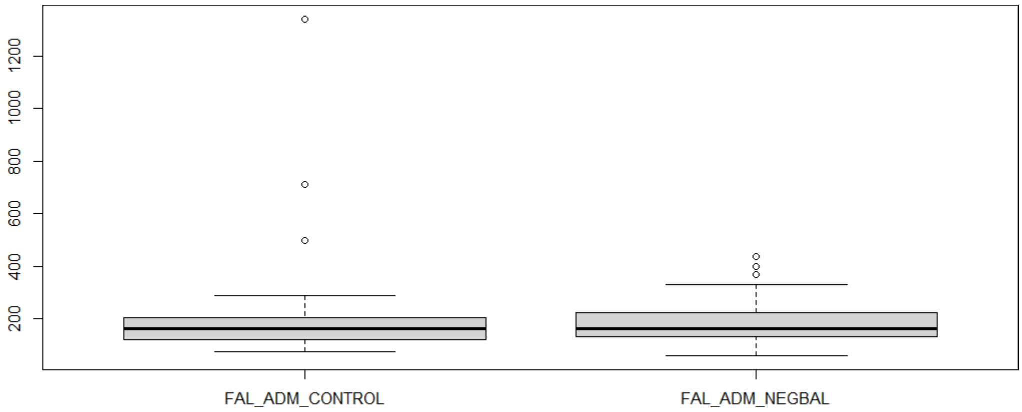

Table S68: Paired differences t−test between FAL ADM CONTROL and FAL ADM NEGBAL.

FAL ADM CONTROL v FAL ADM NEGBAL

t	DF	p−value	95% confidence interval	sample estimates (respectively)
0.65003	64.858	0.518	[-41.06498, 80.69320]	205.1837 185.3696

Table S69: BIT ADM summary.

BIT ADM

	CONTROL GROUP (N = 48)	NEGBAL GROUP (N = 47)
minimum	41.12	5.00
median (IQR)	6.95 (6.09,7.00)	6.70 (5.98,7.00)
mean (sd)	6.68 ± 1.14	6.55 ± 0.74
maximum	12.00	8.51

Figure S27: Boxplot for BIT ADM.

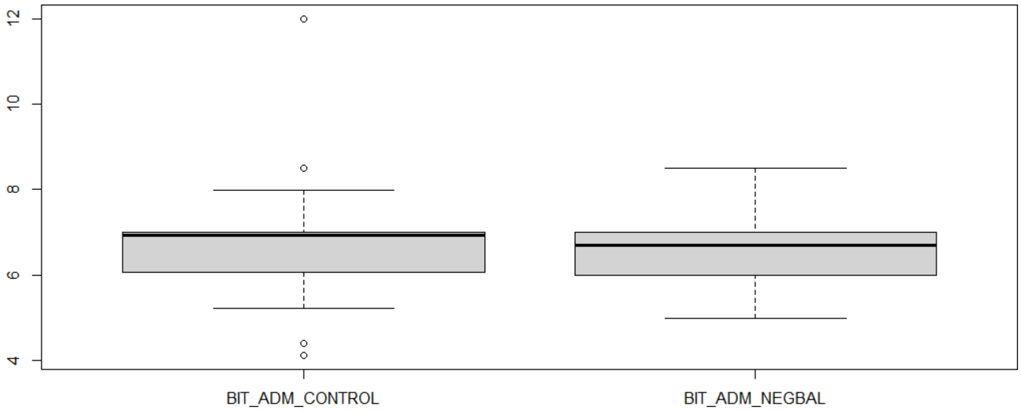

Table S70: Paired differences t−test between BIT ADM CONTROL and BIT ADM NEGBAL.

**BIT ADM CONTROL v BIT ADM NEGBAL**				
t	DF	p−value	95% confidence interval	sample estimates (respectively)
0.65802	81.149	0.5124	[-0.2624909, 0.5219146]	6.683542 6.553830

Table S71: BID ADM summary.

**BID ADM**		
	CONTROL GROUP (N = 49)	NEGBAL GROUP (N = 45)
minimum	0.89	0.39
median (IQR)	1.40 (1.23,1.59)	1.40 (1.25,2.00)
mean (sd)	1.73 ± 1.11	2.44 ± 4.49
maximum	6.00	31.00

Figure S28: Boxplot for BID ADM.

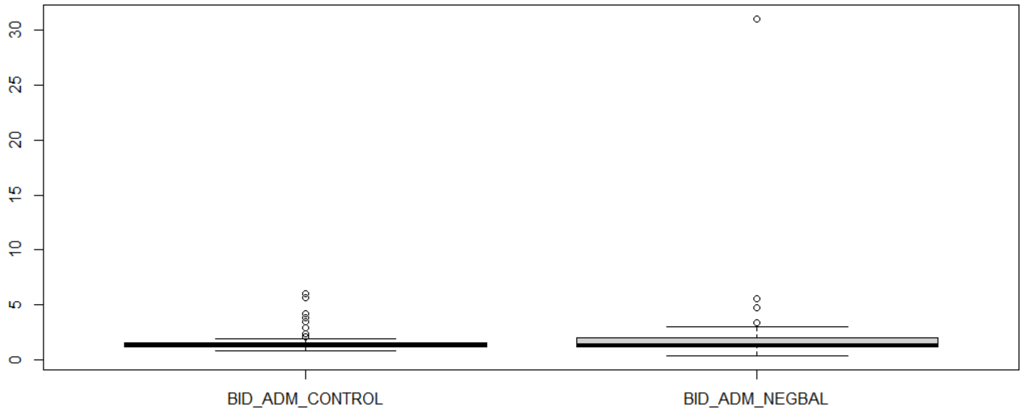

Table S72: Paired differences t−test between BID ADM CONTROL and BID ADM NEGBAL.

**BID ADM CONTROL v BID ADM NEGBAL**				
t	DF	p−value	95% confidence interval	sample estimates (respectively)
-1.0341	48.943	0.3062	[-2.0943874, 0.6712853]	1.732449 2.444000

Table S73: Avergage value, maximum, minimum and standard deviation of selected characteristics for the whole population.

Characteristic	average	maximum	minimum	standard deviation
AGE	59.66	91	22	13.62
WEIGHT	87.26	136	50	18.6
BMI	30.09	42	19	5.16
APACHE II	8.01	21	3	3.26
HR	83.53	120	53	15.01
SBP	127.8	223	93	19.24
DBP	77.47	115	44	12.4
TEMP	36.53	39	35	0.86
RR	24.91	40	13	4.58
pO_2 admission	73.39	149.6	43	18.89
PaFiO_2 admission	164.2	297.9	54	70.21
pCO_2 admission	33.28	50	17	5.15
HCO_3 admission	21.89	31	12	3.06
CREAT admission	9.01	15.3	5.2	2.22
LYMP admission (n = 115)	959.7	5773	122	665.01
TROPO (n = 87)	10.4	41	0.43	8.35
BNP (n = 76)	224.8	873	5	235.12
TGO (n = 96)	49.58	232	12	37.72
TGP (n = 96)	57.25	460	10	68.04
CT score admission (n = 112)	15.72	25	5	4.63
DIAM VCS (n = 111)	17.71	25.4	10.4	2.97
HTO	39.79	52	25	4.91

Table S74: Percentages of age ranges for the whole population and for CONTROL and NEGBAL groups and $p-$value of the comparison CONTROL v NEGBAL.

Age range	No. (%) whole population	No. (%) CONTROL	No. and % NEGBAL	$p-$value
AGE (18-49)	32 (27.59)	15 (25.86)	17 (29.31)	0.6767
AGE (50-64)	40 (34.48)	22 (37.93)	18 (31.03)	0.0158
AGE ($\geq$ 65)	44 (37.93)	21 (36.21)	23 (39.66)	0.00454

Table S75: Proportion of whole population for selected categorical variables.

Characteristic	yes No.(%)	no No.(%)
OBESITY	59 (50.86)	57 (49.14)
HTA	47 (40.52)	69 (59.48)
DBT	30 (25.86)	86 (74.14)
EPOC (n = 115)	20 (17.39)	95 (82.61)
Vaccine (1st dose)	52 (44.83)	64 (55.17)
Vaccine (2nd dose)	27 (23.28)	89 (76.72)
