# Supplementary material for: Lung Injury in COVID-19 Has Pulmonary Edema as an Important Component and Treatment with Furosemide and Negative Fluid Balance (NEGBAL) Decreases Mortality
Source: J Clin Med. 2023 Feb 15;12(4):1542. doi: 10.3390/jcm12041542 (PMC9966668; doi:10.3390/jcm12041542)
Supplement: Supplementary file 1 [file jcm-12-01542-s001.zip › Santos JLF et al. Supplement 2.pdf]

Appendix: Supplemental Statistics Nº2

This appendix has been provided by the authors to give readers additional information about their work.

Supplement to: Jose L. Francisco Santos, MD; Patricio Zanardi, MD; Veronica Alo, MD; Vanina Dos Santos, MD; Leonardo Bovone, MD; Marcelo Rodriguez, MD; Federico Magdaleno, MD; Virginia De Langhe, MD; Andrea Villoldo, MD; Romina Martinez Souvielle, MD; Julieta Alconcher, MD; Diego Quiros, MD; Claudio Milicchio, MD and Eduardo Garcia Saiz, MD. **Lung Injury in COVID-19 has Pulmonary Edema as an important component and treatment with Furosemide and Negative Fluid Balance (NEGBAL) decreases mortality.**

Statistical Report
COVID-19 treatment with NEGBAL protocol

Dr. Hernán A. Vivas
Centro Marplatense de Investigaciones Matemáticas (UNMdP - CIC)

Contents

1. **Data source and description**
2. **Statistical model**
3. **Results**

3.1 Intrinsic evolution

Figure S1: Boxplot for PAFI ADM, PAFI D4, PAFI D8 and PAFI DIS for the CONTROL group.
Table S1: Evolution of PAFI CONTROL group
Table S2: Evolution of PAFI NEGBAL group
Table S3: Results for the paired differences between PAFI ADM and PAFI D04, PAFI ADM and PAFI D08 and PAFI ADMand PAFI DIS for the CONTROL group
Table S4: Results for the paired differences between PAFI ADM and PAFI D4, PAFI ADM and PAFI D8 and PAFI ADMand PAFI DIS for the NEGBAL group.
Figure S2: Boxplot for PAFI ADM, PAFI D4, PAFI D8 and PAFI DIS for the NEGBAL group.
Table S5: Evolution of HEMATOCRIT CONTROL group
Table S6: Evolution of HEMATOCRIT NEGBAL group
Table S7: Results for the paired differences between HCT ADM and HCT D4, HCT ADM and HCT D8 and HCT ADM andHCT DIS for the CONTROL group.
Table S8: Results for the paired differences between HCT ADM and HCT D4, HCT ADM and HCT D8 and HCT ADM andHCT DIS for the NEGBAL group.
Figure S3: Boxplot for HCT ADM, HCT D4, HCT D8 and HCT DIS for the CONTROL group.
Figure S4: Boxplot for HCT ADM, HCT D4, HCT D8 and HCT DIS for the NEGBAL group.
Figure S5: Boxplot for CREAT ADM, CREAT D4, CREAT D8 and CREAT DIS for the CONTROL group.
Table S9: Evolution of CREATININE CONTROL group
Table S10: Evolution of CREATININE NEGBAL group
Table S11: Results for the paired differences between CREAT ADM and CREAT D4, CREAT ADM and CREAT D8 andCREAT ADM and CREAT DIS for the CONTROL group.
Table S12: Results for the paired differences between CREAT ADM and CREAT D4, CREAT ADM and CREAT D8 andCREAT ADM and CREAT DIS for the NEGBAL group.
Figure S6: Boxplot for CREAT ADM, CREAT D4, CREAT D8 and CREAT DIS for the NEGBAL group.
Table S13: CTSCORE previous to the treatment and on the day of admission, the fourth, the eight and the twelfth days forthe CONTROL group.
Table S14: CTSCORE previous to the treatment and on the day of admission, the fourth, the eight and the twelfth days forthe NEGBAL group.
Table S 15: Results for the paired differences between CTSCORE PRE and CTSCORE ADM, CTSCORE ADM andCTSCORE D4, CTSCORE ADM and CTSCORE D8 and CTSCORE ADM CTSCORE D12 for the CONTROL group.
Table S 16: Results for the paired differences between CTSCORE PRE and CTSCORE ADM, CTSCORE ADM andCTSCORE D4, CTSCORE ADM and CTSCORE D8 and CTSCORE ADM CTSCORE D12 for the NEGBAL group.
Figure S 7: Boxplot for CTSCORE PRE, CTSCORE ADM, CTSCORE D4, CTSCORE D8 and

CTSCORE D12 for the CONTROL group.
Figure S8: Boxplot for CTSCORE PRE, CTSCORE ADM, CTSCORE D4, CTSCORE D8 and CTSCORE D12 for the NEGBAL group.
Figure S9: Boxplot for SVC PRE, SVC ADM, SVC D4, SVC D8 and SVC D12 for the CONTROL group.
Table S17: SVC previous to the treatment and on the day of admission, the fourth, the eight and the twelfth days for theCONTROL group
Table S18: SVC previous to the treatment and on the day of admission, the fourth, the eight and the twelfth days for theNEGBAL group.
Table S19: Results for the paired differences between SVC PRE and SVC ADM, SVC ADM and SVC D4, SVC ADM andSVC D8 and SVC ADM SVC D12 for the CONTROL group.
Table S20: Results for the paired differences between SVC PRE and SVC ADM, SVC ADM and SVC D4, SVC ADM andSVC D8 and SVC ADM SVC D12 for the NEGBAL group.
Figure S10: Boxplot for SVC PRE, SVC ADM, SVC D4, SVC D8 and SVC D12 for the NEGBAL group.
Table S21: Results for the paired differences of PAFI D4, PAFI D8 and PAFI DIS between groups.
Figure S11: Boxplot for PAFI D4 CONTROL and PAFI D4 NEGBAL.

3.2 Day by day comparison

Table S22: Results for the paired differences of HCT D4, HCT D8 and HCT DIS between groups.
Figure S12: Boxplot for PAFI D8 CONTROL and PAFI D8 NEGBAL.
Figure S13: Boxplot for PAFI DIS CONTROL and PAFI DIS NEGBAL.
Table S23: Results for the paired differences of CREAT D4, CREAT D8 and CREAT DIS between groups.
Figure S14: Boxplot for HCT D4 CONTROL and HCT D4 NEGBAL.
Figure S15: Boxplot for HCT D8 CONTROL and HCT D8 NEGBAL.
Figure S16: Boxplot for HCT DIS CONTROL and HCT DIS NEGBAL.
Figure S17: Boxplot for CREAT D4 CONTROL and CREAT D4 NEGBAL.
Figure S18: Boxplot for CREAT D8 CONTROL and CREAT D8 NEGBAL.
Figure S19: Boxplot for CREAT DIS CONTROL and CREAT DIS NEGBAL
Table S24: Results LEUC ADM CONTROL and LEUC ADM NEGBAL.
Table S25: Results for the paired differences between LEUC ADM CONTROL and LEUC ADM NEGBAL.
Figure S20: Boxplot for LEUC ADM CONTROL and LEUC ADM NEGBAL.
Table S26: Results for the paired differences of CTSCORE ADM, CTSCORE D4, CTSCORE D8 and CTSCORE D12 between groups.
Table S27: Results for the paired differences of SVC ADM, SVC D4, SVC D8 and SVC D12 between groups.
Figure S21: Boxplot for CTSCORE ADM CONTROL and CTSCORE ADM NEGBAL.
Figure S22: Boxplot for CTSCORE D4 CONTROL and CTSCORE D4 NEGBAL.
Figure S23: Boxplot for CTSCORE D8 CONTROL and CTSCORE D8 NEGBAL.
Figure S24: Boxplot for CTSCORE D12 CONTROL and CTSCORE D12 NEGBAL.
Figure S25: Boxplot for SVC ADM CONTROL and SVC ADM NEGBAL.
Figure S26: Boxplot for SVC D4 CONTROL and SVC D4 NEGBAL.
Figure S27: Boxplot for SVC D8 CONTROL and SVC D8 NEGBAL.
Figure S28: Boxplot for SVC D12 CONTROL and SVC D12 NEGBAL.
Figure S29: Boxplot for ACC HYD BAL CONTROL and ACC HYD BAL NEGBAL.
Table S28: Results of Accumulated Hydric Balance of CONTROL group.
Table S29: ACC HYD BAL for survivors of the CONTROL group.
Table S30: ACC HYD BAL for nonsurvivors of the CONTROL group.
Figure S30: Boxplot for HOSP DAYS CONTROL and HOSP DAYS NEGBAL.
Table S31: Results of Accumulated Hydric Balance of NEGBAL group.
Table S32: Results for the paired differences of ACC HYD BAL between groups.
Figure S31: Boxplot for ICU DAYS CONTROL and ICU DAYS NEGBAL.
Figure S32: Boxplot for IMV DAYS CONTROL and IMV DAYS NEGBAL.
Table S33: Results of HOSP DAYS, ICU DAYS and IMV DAYS in CONTROL group.
Table S34: Results of HOSP DAYS, ICU DAYS and IMV DAYS in NEGBAL group.
Table S35: Results for the paired differences of HOSP DAYS, ICU DAYS and IMV DAYS between groups.
Table S36: Result frequencies
Table S37: Pearson's χ^2 test (Yates' continuity correction)
Figure S33: Mosaic plot for the result of the treatment.
Table S38: Results for the paired differences of ACC HYD BAL between survivors and nonsurvivors within groups and in thewhole population.

3.3 Regression

Figure S34: Scatter plot and regression line for ACC HYD BAL CONTROL and PAFI DIS CONTROL, $p =$ 0.007238.
Figure S35: Scatter plot and regression line for ACC HYD BAL NEGBAL and PAFI DIS NEGBAL , $p =$

0.0429.
Figure S36: Stripchart for PAFI v VAC.

3.4 Accumulated hydric balance comparison

Figure S37: Boxplot for ACC HYD BAL CONTROL SUR and ACC HYD BAL CONTROL NONSUR.
Figure S38: Boxplot for ACC HYD BAL NEGBAL SUR and ACC HYD BAL NEGBAL NONSUR.
Figure S39: Boxplot for ACC HYD BAL SUR and ACC HYD BAL NONSUR.

3.5 Attached Dossier

Table S39: Result IMV use
Figure S40: Boxplot for ICU from SSD CONTROL and ICU from SSD NEGBAL

1 Data source and description

We analyzed data from 116 patients treated for COVID-19 disease. A first group of 58 patients was treated with the standardtreatment (CONTROL group) and a second group was treated following the NEGBAL protocol (NEGBAL group). At this stage, we compared data from both populations as to inquire whether there was any statistically significant difference betweenthe evolution of those patients treated with NEGBAL protocol and those treated with the standard treatment.

The data consisted of: PaFiO_2, creatinine (CREAT) and hematocrit (HCT) measurements for at the moment of admission,at days 4 and 8 and at discharge[1]; leukocytes (LEUC) at the moment of admission; accumulated hydric balance after the 8 days (ACC HYD BAL); hospitalization days (DAYS INT), ICU days (ICU DAYS) and days under MV (DAYS MiV); andresult of the treatment (RES, alive or dead); CT score and superior vena cava diameter (CTSCORE and SVC respectively) measurements previous to the treatment, at the moment of admission, at days 4, 8 and 12.

On some variables there were missing values, namely: PAFI D4 CONTROL (2), PAFI D8 CONTROL (13), PAFI D4 NEGBAL (3), PAFI D8 NEGBAL (14), PAFI DIS NEGBAL (4), HCT D4 CONTROL (2), HCT D8 CONTROL (14), HCT D4 CONTROL (3), HCT D8 CONTROL (17), CREAT D4 CONTROL (2), CREAT D8 CONTROL (12), CREAT D4 NEGBAL (3), CREAT D4 NEGBAL (16), CT SCORE PRE CONTROL (34), CT SCORE ADM CONTROL (4), CT SCORE D4 CONTROL (37), CT SCORE D8 CONTROL (41), CT SCORE D12 CONTROL (38), CT SCORE PRE NEGBAL (39), CT SCORE D4 NEGBAL (3), CT SCORE D8 NEGBAL (18), CT SCORE D12 NEGBAL (50), SVC PRE CONTROL (34), SVC ADM CONTROL (4), SVC D4 CONTROL (37), SVC D8 CONTROL (41), SVC D12 CONTROL (38), SVC PRE NEGBAL (39), SVC ADM NEGBAL (1), SVC D4 NEGBAL (3), SVC SCORE D8 NEGBAL (19), SVC SCORE D12 NEGBAL (50), ACC HYD BAL CONTROL (9).

2 Statistical model

Five types of analyses were performed:

1. an intrinsic analysis via paired differences t tests regarding the evolution of the variables PaFiO_2, CREAT (creatinine)and HCT (hematocrit) at the moment of admission, days 4 and 8 and discharge and CTSCORE and VSC previous to the treatment, at the moment of admission, and at days 4, 8 and 12 *for the same group* ;
2. a *comparative* analysis via unpaired differences t tests regarding of the same variables as above (as well as the variable LEUC at the moment of admission) but comparing for each day the values of both groups, together with a comparison (again using unpaired difference t tests) of the variables ACC HYD BAL and hospitalization days between groups anda Pearson's χ^2 test for result;
3. a simple regression model to asses the correlation between PaFiO_2 and ACC HYD BAL in both groups:
$$\text{PaFi}O_2 \sim \beta_0 + \beta_1 \text{ACC HYD BAL}; \tag{1}$$
4. a multivariate regression model to asses the correlation between PaFiO_2 and ACC HYD BAL and vaccination (VAC)for the whole population:
$$\text{PaFi}O_2 \sim \beta_0 + \beta_1 \text{ACC HYD BAL} + \beta_1 \text{VAC}; \tag{2}$$
5. a paired differences t tests comparing the values of the variable ACC HYD BAL for survivors and non survivors withineach group and for the whole population.

3 Results

We divide the results in subsections according to the analyses detailed in the previous paragraph: intrinsic evolution, day byday comparison and regression.

---

[1]All the data supplied consisted on the same measurements for both groups, except for the missing values.

Figure S1: Boxplot for PAFI ADM, PAFI D4, PAFI D8 and PAFI DIS for the CONTROL group.

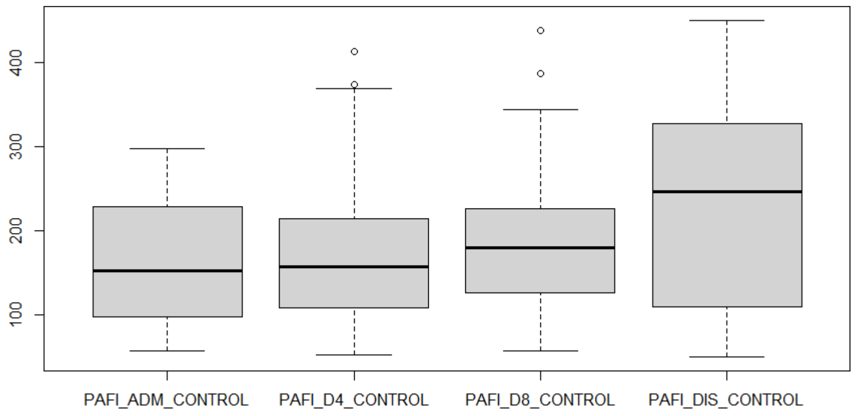

3.1 Intrinsic evolution

Tables 1 and 2 contain, respectively, the data of PaFiO_2 for the CONTROL and NEGBAL groups at the moment of admission, days 4 and 8 and discharge; the results for the paired differences between PAFI ADM and PAFI D4, PAFI ADM and PAFI D8 and PAFI ADM and PAFI DIS can be found respectively in Tables 3 and 4 while the corresponding boxplots are in Figures 1 and 2. We point out the presence of outliers: PAFI D4 CONTROL (2), PAFI D8 CONTROL (2), PAFI DIS NEGBAL (6).

Tables 5 and 6 contain, respectively, the data of HCT for the CONTROL and NEGBAL groups at the moment of admission, days 4 and 8 and discharge; the results for the paired differences between HCT ADM and HCT D4, HCT ADM and HCT D8 and HCT ADM and HCT DIS can be found respectively in Tables 7 and 8 while the corresponding boxplots are in Figures 3 and 4. We point out the presence of outliers: HCT ADM CONTROL (3), HCT D4 CONTROL (2),HCT D8 CONTROL (1), HCT DIS CONTROL (1), HCT ADM NEGBAL (1), HCT D4 NEGBAL (2), HCT D8 NEGBAL(1), HCT DIS NEGBAL (1).

Tables 9 and 10 contain, respectively, the data of CREAT for the CONTROL and NEGBAL groups at the moment of admission, days 4 and 8 and discharge; the results for the paired differences between CREAT ADM and CREAT D4, CREAT ADM and CREAT D8 and CREAT ADM and CREAT DIS can be found respectively in Tables 11 and 12 while the corresponding boxplots are in Figures 5 and 6. We point out the presence of outliers: CREAT ADM CONTROL (6), CREAT D4 CONTROL (3), CREAT D8 CONTROL (3), CREAT DIS CONTROL (9), CREAT D4 NEGBAL (2), CREAT D8 NEGBAL (1), CREAT DIS NEGBAL (7).

The data of CTSCORE for the CONTROL and NEGBAL groups can be found, respectively, in Tables 13 and 14 and Figures 7 and 8 display the boxplots of this data for each group separately. Tables 15 and 16 display the result of the *t* test comparison for CTSCORE previous to the treatment and on the admission day and the admission day compared to days 4,8 and 12 for each group (CONTROL and NEGBAL respectively).

The data of SVC for the CONTROL and NEGBAL groups can be found, respectively, in Tables 17 and 18 and Figures 9 and 10 display the boxplots of this data for each group separately. Tables 19 and 20 display the result of the *t* test comparison for SVC previous to the treatment and on the admission day and the admission day compared to days 4, 8 and 12 for each group (CONTROL and NEGBAL respectively).

Table S1: Evolution of PAFI CONTROL group

	PAFI-ADM-CONTROL	PAFI-D04-CONTROL	PAFI-D08-CONTROL	PAFI-DIS-CONTROL
1	131.67	122.00	158.00	87.00
2	100.00	106.25	318.93	357.00
3	198.33	212.50	84.44	108.00
4	277.78		-	211.00
5	75.00	115.00	249.60	404.00
6	277.14	317.14	-	422.00
7	172.50	157.50	-	314.00
8	100.00	253.12	300.00	328.00
9	113.75	215.62	271.43	304.00
10	228.57	187.50	-	371.00
11	277.78	314.29	-	314.00
12	185.00	192.50	140.00	188.00
13	152.50	92.57	181.00	250.00
14	82.50	76.67	146.67	56.00
15	106.00	280.00	234.00	97.00
16	149.75	81.11	237.50	243.00
17	201.67	111.25	98.57	148.00
18	108.67	52.00	83.00	142.00
19	172.50	148.80	126.15	320.00
20	166.00	156.80	122.75	331.25
21	268.61	172.50	305.71	451.00
22	235.62	92.86	116.00	70.00
23	160.00	75.00	70.00	218.00
24	140.00	370.00	210.00	162.00
25	227.78	160.00	180.00	282.00
26	71.11	134.55	133.33	50.00
27	221.88	116.67	211.11	312.00
28	80.00	71.56	63.50	200.00
29	245.36	413.21	226.67	256.66
30	80.29	164.00	188.40	73.10
31	271.43	151.67	188.00	58.00
32	68.89	297.78	202.50	357.00
33	70.00	210.00	154.00	161.00
34	82.86	208.33	-	175.00
35	279.17	143.33	156.00	100.00
36	270.83	147.22	-	205.00
37	86.67	74.00	72.86	329.00
38	143.33	293.33	-	290.00
39	57.00	62.00	57.00	67.00
40	278.57	212.50	-	342.00
41	69.00	121.67	-	117.00
42	78.57	77.12	205.00	237.50
43	89.33	92.20	128.00	91.43
44	181.00	309.17	387.14	387.00
45	214.29	374.76	439.05	364.29
46	150.25	171.14	163.89	235.70
47	147.14	328.57	-	333.00
48	97.43	246.67	130.00	323.30
49	240.00	309.52	-	314.00
50	99.12	110.43	212.50	417.00
51	147.14	122.83	344.64	317.92
52	297.92	89.29	94.60	317.00
53	79.57	111.00	78.00	110.00
54	190.94	161.17	183.25	379.00
55	260.71	158.17	189.60	102.00
56	152.50	192.50	240.00	271.00
57	260.36	102.00	168.00	60.00
58	187.00	-	-	96.40
Mean	164.81	175.7	183.4	235.0
Median	152.50	157.2	180.0	246.5
Standard deviation	73.64	90.59	87.03	116.48

Table S2: Evolution of PAFI NEGBAL group

	PAFI ADM NEGBAL	PAFI D04 NEGBAL	PAFI D08 NEGBAL	PAFI DIS NEGBAL
1	60.00	224.00	195	78
2	61.60	247.50	366	463
3	65.00	142.50	340	309
4	188.00	460.71	-	347
5	96.25	81.25	218	323
6	110.00	328.12	-	371.43
7	61.25	125.00	255	342
8	100.00	130.00	367	300
9	177.78	400.00	-	400
10	86.00	111.11	117	251.7
11	120.00	205.00	407	404
12	121.67	233.33	323	352
13	101.25	253.57	384	380
14	185.00	314.29	-	314
15	145.00	268.75	341	302.3
16	197.50	341.67	-	366
17	102.00	105.00	225	103.1
18	160.00	190.00	189	257.8
19	104.29	376.19	499	495
20	162.50	375.00	314	361
21	184.38	287.50	-	342
22	172.22	352.38	395.7	395.7
23	225.00	304.76	297	376.6
24	258.33	401.90	-	417.6
25	146.00	94.00	90	77
26	231.25	322.92	318	300
27	126.25	240.00	355	318
28	203.12	253.93	250	309
29	140.00	-	349	347
30	90.00	137.14	307	338
31	178.12	251.07	322	375
32	250.00	333.33	376	380
33	259.38	-	-	353
34	246.43	262.14	-	456
35	191.67	258.33	367	309
36	58.00	61.25	82	100
37	177.50	132.86	50	118
38	256.25	309.52	333	432
39	272.22	438.10	364	361
40	281.56	189.67	278	312.6
41	197.25	202.22	215	215
42	209.69	388.57	-	411
43	207.14	-	-	321
44	171.88	208.33	289	361
45	264.29	433.33	-	376
46	85.71	254.44	367	495
47	128.00	193.80	268	378
48	89.33	164.36	-	127.5
49	154.50	235.00	319	346
50	103.33	133.00	366	342
51	270.00	361.90	-	411
52	54.00	81.25	108	408.7
53	84.20	217.40	353	471.9
54	231.11	237.19	190	338
55	228.21	335.36	487	487
56	256.88	263.93	257	293
57	162.50	230.00	253	290.4
58	235.71	241.79	233	480
Mean	163.6	249.56	290.4	344.0
Median	167.2	247.50	316.0	352.5
Standard deviation	67.25	100.5	101.04	97.63

Table S3: Results for the paired differences between PAFI ADM and PAFI D04, PAFI ADM and PAFI D08 and PAFI ADMand PAFI DIS for the CONTROL group

	t	DF	p−value	95 % confidence interval
PAFI_ADM_CONTROL and PAFI_D4_CONTROL	-0.97452	55	0.3341	[-40.78967, 14.09866]
PAFI_ADM_CONTROL and PAFI_D8_CONTROL	-1.9113	44	0.06249	[61.383470, 1.626253]
PAFI_ADM_CONTROL and PAFI_FINAL_CONTROL	-4.1863	57	9.928e-05	[-103.70732, -36.59521]

Table S4: Results for the paired differences between PAFI ADM and PAFI D4, PAFI ADM and PAFI D8 and PAFI ADMand PAFI DIS for the NEGBAL group.

	t	DF	p−value	95 % confidence interval
PAFI_ADM_NEGBAL and PAFI_D4_NEGBAL	-8.2238	54	4.302e-11	[-109.58259, -66.62487]
PAFI_ADM_NEGBAL and PAFI_D8_NEGBAL	-8.1042	43	3.376e-10	[-174.2546, -104.8107]
PAFI_ADM_NEGBAL and PAFI_DIS_NEGBAL	-12.12	53	< 2.2e-16	[-206.8090, -148.0772]

Figure S2: Boxplot for PAFI ADM, PAFI D4, PAFI D8 and PAFI DIS for the NEGBAL group.

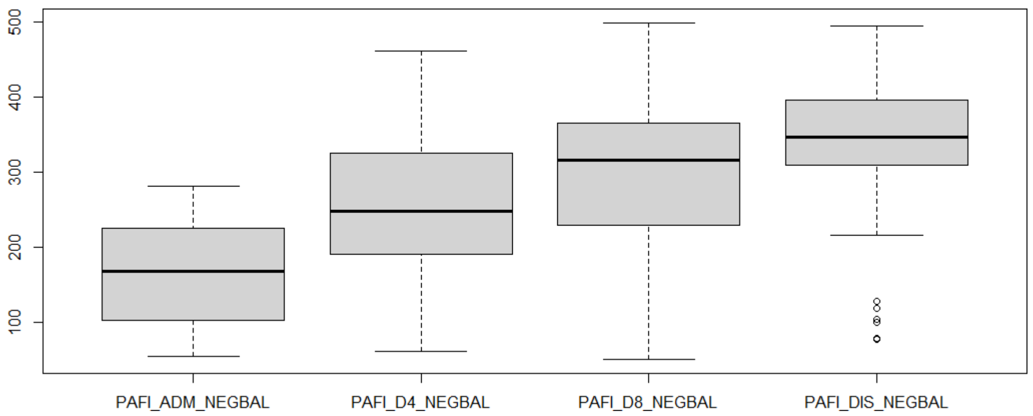

Table S5: Evolution of HEMATOCRIT CONTROL group

	HCT-ADM CONTROL	HCT-D4 CONTROL	HCT-D8 CONTROL	HCT-DIS CONTROL
1	37.00	37.00	36.20	39.30
2	42.00	38.00	40.30	36.20
3	39.00	34.60	34.20	26.60
4	37.10	-	-	44.7
5	44.00	45.00	40.30	43.90
6	47.50	44.90	-	44.9
7	26.00	29.00	-	26.7
8	32.00	34.00	32.80	32.80
9	46.00	42.00	-	43.70
10	40.00	38.00	-	38.20
11	40.70	39.00	-	39.30
12	28.00	28.00	28.6	28.7
13	34.30	32.60	35.5	23.9
14	45.00	42.00	36.1	33.70
15	45.00	40.00	37.80	26.2
16	38.30	39.00	39.6	40.4
17	35.70	36.00	36.30	21.7
18	41.00	39.00	39.20	40.9
19	38.80	32.20	31.5	41.6
20	45.00	44.60	41.2	39.6
21	41.00	41.00	42.1	41.1
22	43.00	43.00	41.8	38.4
23	44.00	40.00	40.9	37.4
24	40.90	41.00	32.9	36.6
25	36.00	36.00	33.70	34.70
26	52.00	41.00	44.6	32.80
27	38.00	37.00	38.20	39.4
28	44.70	39.60	40.80	40.5
29	40.10	42.20	44.2	40.6
30	39.20	35.30	36.6	23.3
31	46.00	43.00	36.1	30.1
32	34.00	31.00	32.20	26.1
33	41.00	44.00	34.80	19.90
34	42.00	46.00	-	47.4
35	47.00	40.00	38.80	39.70
36	40.00	37.00	-	36.20
37	41.00	42.00	45.3	39.1
38	43.00	42.00	-	42.3
39	47.00	46.00	46.6	36.5
40	36.00	42.00	-	36.9
41	52.00	53.00	-	45.7
42	43.60	41.40	43.9	44
43	39.10	42.60	38.70	38.70
44	36.10	45.40		48.1
45	38.00	39.80	31.9	30.2
46	38.90	40.80	40.4	36.9
47	33.00	30.00	-	32.80
48	38.30	35.50	37.10	30.80
49	41.70	40.40	42.40	42.40
50	45.30	36.10	41.20	41.90
51	41.60	41.20	41.50	40.90
52	46.50	48.80	46.20	42.50
53	38.10	31.20	33.40	35.80
54	42.50	40.40	45.40	43.20
55	39.00	26.40	24.80	24.30
56	40.00	35.00	36.90	38.10
57	42.60	40.30	41.30	29.90
58	27.40	-	-	25.40
Mean	40.38	39.15	38.5	38.2
Median	40.80	40.00	38.8	36.4
Standard deviation	5.22	5.21	4.67	6.99

Table S5: Evolution of HEMATOCRIT NEGBAL group

	HCT_ADM_NEGBAL	HCT_D4_NEGBAL	HCT_D8_NEGBAL	HCT_DIS_NEGBAL
1	44.00	42.00	47.6	43.50
2	25.00	30.00	28.8	31.00
3	37.00	44.00	43.7	38.90
4	29.30	40.00	-	43.10
5	42.70	45.00	43.9	42.40
6	43.00	43.00	-	43.00
7	35.00	34.00	39.30	34.40
8	41.00	44.00	42.7	41.20
9	38.00	40.00	-	40.60
10	37.40	37.00	40.8	37.30
11	38.00	41.00	40.70	40.70
12	35.00	38.00	37.80	36.60
13	45.00	45.00	43.70	43.70
14	39.00	43.00	-	40.40
15	35.70	38.00	35.6	37.70
16	41.00	53.00	-	45.60
17	38.30	46.00	39.6	43.50
18	34.90	40.00	37.9	34.20
19	31.30	32.00	30.3	30.50
20	40.00	42.00	44.4	44.40
21	34.00	43.00	-	44.30
22	45.00	45.00	-	47.70
23	29.30	42.00	-	31.50
24	35.60	46.00	-	35.10
25	44.90	49.90	52.7	51.20
26	42.00	47.80	46.6	43.20
27	37.00	42.50	40.1	40.30
28	36.60	37.90	41.3	41.00
29	38.60	50.10	49.3	49.30
30	35.70	38.00	37.1	36.40
31	38.00	43.50	42.1	34.20
32	41.40	41.00	46.7	48.90
33	37.20	-	-	38.10
34	45.00	45.40	-	47.50
35	43.00	46.00	50.2	45.70
36	44.00	45.00	49.2	54.30
37	32.00	34.00	42.2	41.10
38	42.00	41.00	45.6	43.20
39	42.00	44.00	40.4	40.00
40	40.30	41.90	45.6	41.00
41	45.00	44.80	-	44.40
42	44.80	49.40	-	50.30
43	42.00	-	-	44.20
44	44.60	50.00	47.6	44.90
45	40.00	49.00		47.00
46	38.00	36.30	37.30	36.60
47	43.60	50.30	54	32.40
48	41.80	42.90		50.80
49	39.20	48.60	41.9	36.60
50	41.00	50.00	47.5	42.70
51	45.00	43.00	-	43.10
52	42.00	44.00	42	24.90
53	42.40	53.30	42.9	42.50
54	38.20	-	39.1	39.60
55	31.80	32.70	39.9	33.40
56	41.30	45.30	42	33.90
57	42.00	44.00	40.5	37.70
58	36.90	46.10	41.8	40.40
Mean	39.20	43.10	42.54	40.90
Median	40.00	43.50	42.05	41.05
Standard deviation	4.54	5.21	5.23	5.79

Table S7: Results for the paired differences between HCT_ADM and HCT_D4, HCT_ADM and HCT_D8 and HCT_ADM and HCT_DIS for the CONTROL group.

	t	DF	*p*−value	95 % confidence interval
HCT_ADM_CONTROL and HCT_D4_CONTROL	2.9498	55	0.004663	[0.4878045, 2.5550527]
HCT_ADM_CONTROL and HCT_D8_CONTROL	4.2894	43	0.0001028	[1.320401, 3.720508]
HCT_ADM_CONTROL and HCT_DIS_CONTROL	4.69	57	1.75e-05	[2.355380, 5.865309]

Table S8: Results for the paired differences between HCT_ADM and HCT_D4, HCT_ADM and HCT_D8 and HCT_ADM and HCT_DIS for the NEGBAL group.

	t	DF	*p*−value	95 % confidence interval
HCT_ADM_NEGBAL and HCT_D4_NEGBAL	-7.2295	54	1.746e-09	[-4.976901, -2.815826]
HCT_ADM_NEGBAL and HCT_D8_NEGBAL	-6.9727	40	2.047e-08	[-4.517636, -2.487242]
HCT_ADM_NEGBAL and HCT_DIS_NEGBAL	-2.5765	57	0.01259	[-3.0120399, -0.3776153]

Figure S3: Boxplot for HCT_ADM, HCT_D4, HCT_D8 and HCT_DIS for the CONTROL group.

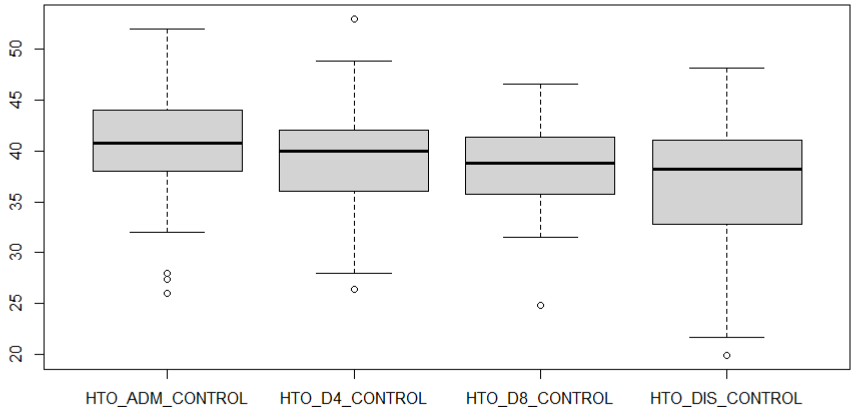

Figure S4: Boxplot for HCT ADM, HCT D4, HCT D8 and HCT DIS for the NEGBAL group.

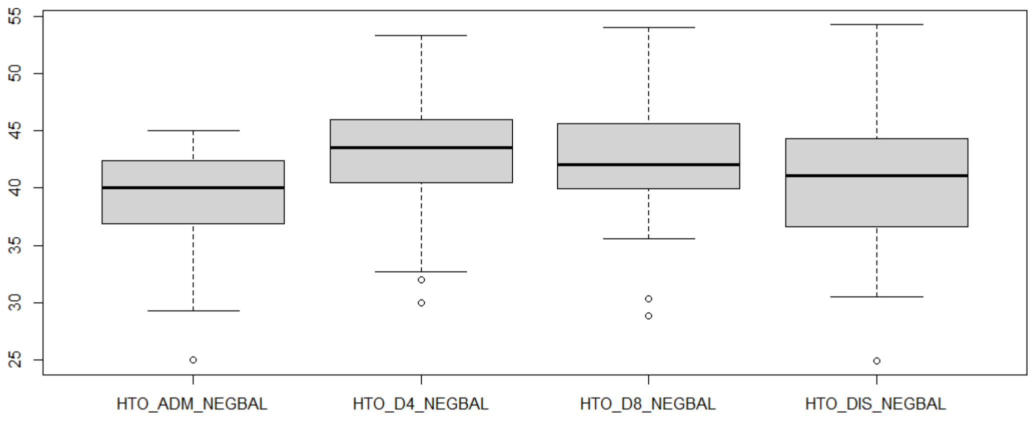

Figure S5: Boxplot for CREAT ADM, CREAT D4, CREAT D8 and CREAT DIS for the CONTROL group.

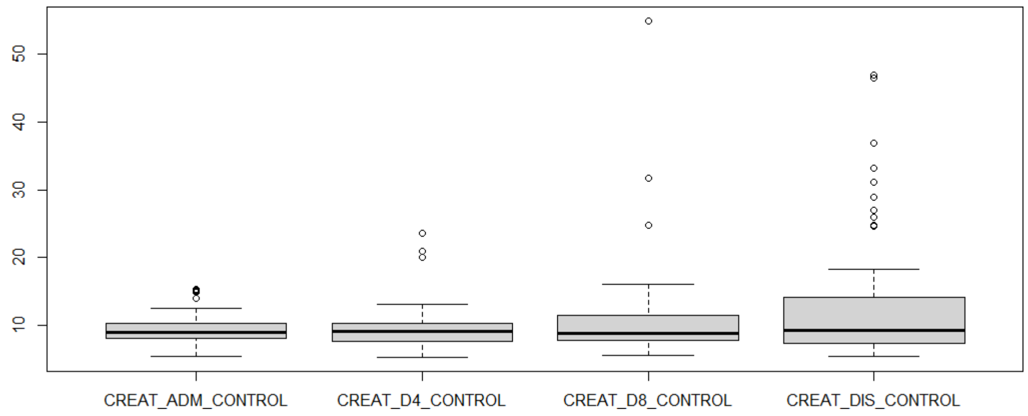

Table S9: Evolution of CREATININE CONTROL group

	CREAT_ADM_CONTROL	CREAT_D4_CONTROL	CREAT_D8_CONTROL	CREAT_DIS_CONTRO
1	10.00	11.00	10.00	6.60
2	8.90	7.40	6.80	8.50
3	14.80	10.90	9.40	9.10
4	15.10	-	-	16.20
5	9.00	10.00	8.50	11.40
6	9.30	9.40	-	9.40
7	11.50	11.50	-	13.90
8	15.30	12.50	14.10	14.10
9	11.00	10.30	11.00	10.50
10	9.40	9.50	-	10.10
11	8.40	9.80	-	9.80
12	7.10	5.20	7.40	25.90
13	9.70	13.00	16.00	27.00
14	7.00	20.00	31.70	28.90
15	9.00	9.00	12.60	36.90
16	6.90	8.30	8.10	7.00
17	9.30	7.60	7.30	7.00
18	8.70	6.70	7.80	7.30
19	6.20	8.30	7.90	7.30
20	14.00	13.10	10.90	9.50
21	8.40	8.50	8.90	6.10
22	9.90	7.20	7.70	8.70
23	8.50	9.80	9.60	10.40
24	12.40	9.30	8.30	14.90
25	9.80	9.90	9.30	10.40
26	11.50	8.50	12.40	31.10
27	8.30	7.00	8.30	8.20
28	8.10	8.50	8.20	9.80
29	10.10	9.90	8.60	8.80
30	11.10	11.80	12.40	14.30
31	15.00	23.60	55.00	46.50
32	10.40	9.80	12.80	9.00
33	10.30	11.90	12.10	18.30
34	8.90	9.20	-	10.30
35	11.00	9.30	11.90	24.60
36	10.60	8.50	-	8.00
37	7.10	8.40	8.80	9.10
38	7.80	6.50	-	7.20
39	8.00	9.20	8.30	9.10
40	6.80	6.00	-	6.30
41	14.90	20.90	-	47.00
42	7.20	7.30	8.20	7.80
43	8.90	10.50	24.80	24.80
44	6.10	6.90	6.30	6.30
45	6.30	7.50	7.60	9.90
46	6.50	8.00	8.20	8.50
47	7.40	6.20	-	6.40
48	8.10	10.20	7.00	6.90
49	10.10	7.60	8.80	8.80
50	8.10	6.60	5.50	8.30
51	8.90	9.10	9.30	9.60
52	7.90	7.70	6.70	6.10
53	5.40	5.20	7.80	5.80
54	10.10	7.30	7.00	6.50
55	9.00	12.60	11.00	33.20
56	8.40	8.70	11.40	5.30
57	9.60	8.10	10.60	9.30
58	8.20	-	-	6.00
Mean	9.41	9.58	11.14	13.17
Median	8.95	9.05	8.80	9.20
Standard deviation	2.38	3.44	8.02	9.88

Table S10: Evolution of CREATININE NEGBAL group

	CREAT ADM NEGBAL	CREAT D4 NEGBAL	CREAT D8 NEGBAL	CREAT DIS NEGBAL
1	13.10	11.00	11.2	33.20
2	6.00	4.70	-	4.40
3	8.20	8.90	7.9	7.20
4	6.70	11.00		11.20
5	8.20	7.10	9.1	8.90
6	6.90	7.10	-	7.30
7	6.10	7.10	12.2	8.50
8	11.00	8.10	10.4	10.10
9	6.00	8.10	9.4	9.40
10	10.00	7.10	11.3	7.70
11	9.10	8.80	-	8.30
12	10.30	10.10	11.5	10.40
13	9.40	11.30	11	11.00
14	9.00	10.60	-	10.10
15	11.10	13.00	10.8	10.00
16	10.00	13.20	-	10.70
17	7.00	9.30	8.20	12.50
18	9.00	12.60	13.7	10.40
19	7.10	8.00	6.5	6.30
20	9.50	9.80	10.20	10.20
21	12.80	16.00	-	15.20
22	7.60	9.00	-	10.00
23	8.00	11.40	16	6.00
24	6.00	6.60	-	7.10
25	10.20	12.40	18.3	31.80
26	8.00	9.80	8.4	6.10
27	7.50	10.30	8	6.80
28	8.20	8.30	10.9	8.30
29	6.40	10.20	8.1	8.10
30	7.70	6.10	6.5	5.90
31	11.90	11.10	12.1	11.20
32	8.00	8.20	8.80	11.80
33	6.10	-	-	5.80
34	12.10	21.10	-	19.30
35	9.80	13.00	9.5	8.80
36	11.00	16.00	19	18.60
37	11.20	18.70	19.2	31.70
38	6.20	7.80	7.2	6.50
39	6.90	9.50	8.1	8.00
40	8.40	9.40	8.6	8.50
41	6.50	10.70	9.4	9.40
42	6.60	10.80	-	11.30
43	11.00	-	-	12.00
44	6.40	9.80	6.5	6.80
45	8.00	11.00	-	12.30
46	8.30	14.20	16	10.10
47	10.20	12.70	15.7	9.50
48	9.80	11.40	-	45.50
49	8.20	7.70	6.2	7.00
50	12.00	15.80	14.5	12.00
51	5.20	8.10	-	8.30
52	8.00	13.00	24.5	4.20
53	12.30	24.60	10.3	12.30
54	7.50	-	9	8.00
55	10.60	19.70	18.5	38.40
56	10.50	18.30	13.8	11.20
57	9.00	15.00	12	8.50
58	9.70	7.00	7.5	6.70
Mean	8.75	11.12	11.33	11.67
Median	8.25	10.30	10.35	9.45
Standard deviation	2.01	3.98	4.16	8.23932

Table S11: Results for the paired differences between CREAT_ADM and CREAT_D4, CREAT_ADM and CREAT_D8 and CREAT_ADM and CREAT_DIS for the CONTROL group.

	t	DF	p−value	95 % confidence interval
CREAT_ADM_CONTROL and CREAT_D4_CONTROL	-0.68831	55	0.4942	[-0.9988438, 0.4881295]
CREAT_ADM_CONTROL and CREAT_D8_CONTROL	-1.6794	45	0.1	[-4.0591368, 0.3678324]
CREAT_ADM_CONTROL and CREAT_DIS_CONTROL	-3.132	57	0.00274	[-6.170210, -1.357376]

Table S12: Results for the paired differences between CREAT_ADM and CREAT_D4, CREAT_ADM and CREAT_D8 and CREAT_ADM and CREAT_DIS for the NEGBAL group.

	t	DF	p−value	95 % confidence interval
CREAT_ADM_NEGBAL and CREAT_D4_NEGBAL	-5.6302	54	6.632e-07	[-3.173263, -1.506737]
CREAT_ADM_NEGBAL and CREAT_D8_NEGBAL	-4.1396	41	0.0001689	[-3.592123, -1.236448]
CREAT_ADM_NEGBAL and CREAT_DIS_NEGBAL	-2.9788	57	0.004246	[-4.881223, -0.956708]

Figure S6: Boxplot for CREAT_ADM, CREAT_D4, CREAT_D8 and CREAT_DIS for the NEGBAL group.

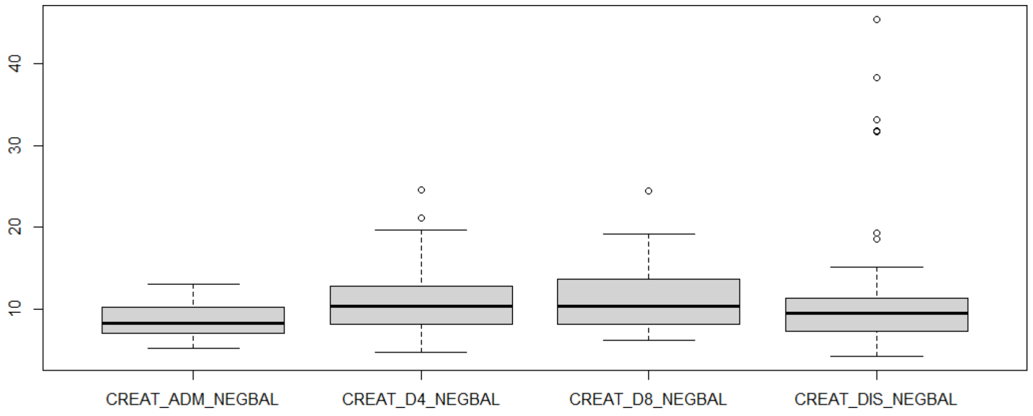

Table S13: CTSCORE previous to the treatment and on the day of admission, the fourth, the eight and the twelfth days forthe CONTROL group.

	CTSCORE PRE CONTROL	CTSCORE ADM CONTROL	CTSCORE D4 CONTROL	CTSCORE D8 CONTROL	CTSCORE D12 CONTROL
1	-	17.00	-	-	8.00
2	-	19.00	15.00	-	-
3	-	21.00		22.00	22.00
4	11.00	13.00	-	-	-
5	22.00	24.00	24.00	-	-
6		10.00	-	-	-
7	14.00	14.00	-	-	-
8	8.00	8.00	-	-	-
9	-	-	12.00	-	-
10	-	5.00	-	-	-
11	-	12.00	10.00	-	-
12	4.00	20.00	-	-	-
13	8.00	13.00	-	-	10.00
14	0.00	10.00	-	-	24.00
15	-	25.00	-	-	17.00
16	10.00	15.00	15.00		13.00
17	-	-	-	11.00	-
18	10.00	14.00	-	22.00	-
19	10.00	16.00	-	11.00	13.00
20	-	13.00	13.00	-	11.00
21	-	13.00	15.00	11.00	-
22	-	19.00		21.00	23.00
23	-	14.00	13.00	-	12.00
24	7.00	15.00	-	-	14.00
25	-	16.00	20.00	-	
26	-	9.00	-	-	-
27	13.00	20.00	18.00	-	-
28	5.00	17.00	20.00	20.00	23.00
29	-	15.00	14.00	13.00	15.00
30	-	16.00	-	24.00	-
31	16.00	22.00	-	-	-
32	15.00	18.00	-	-	11.00
33	-	19.00	-	14.00	-
34	-	19.00	20.00	-	-
35	6.00	13.00	-	-	-
36	18.00	18.00	-	-	-
37	11.00	20.00	-	20.00	-
38	9.00	15.00	10.00	-	-
39		19.00	24.00	-	-
40	13.00	24.00	20.00	-	-
41	-	-	-	-	-
42	-	20.00	21.00	20.00	-
43	-	14.00	-	-	-
44	-	17.00	-	-	-
45	12.00	14.00	11.00	-	-
46	-	17.00		17.00	17.00
47	-	14.00	14.00	-	-
48	-	19.00	-	20.00	16.00
49	9.00	11.00	-	4.00	-
50	1.00	16.00	-	-	14.00
51		21.00	18.00	8.00	-
52	-	-	-	-	-
53	-	11.00	-	-	-
54	-	16.00	-	-	17.00
55	-	12.00	-	-	13.00
56	-	11.00	-	-	
57	8.00	15.00	19.00	20.00	19.00
58	-	17.00	-	-	-
Mean	10.00	15.83	16.48	16.35	15.60
Median	10.00	16.00	15.00	20.00	14.50
Standard deviation	5.06	4.14	4.31	5.79	4.64

Table S14: CTSCORE previous to the treatment and on the day of admission, the fourth, the eight and the twelfth days forthe NEGBAL group.

	CTSCORE PRE NEGBAL	CTSCORE ADM NEGBAL	CTSCORE D4 NEGBAL	CTSCORE D8 NEGBAL	CTSCORE D12 NEGBAL
1	6	21.00	-	7	-
2	-	13.00	8	-	4
3	12	20.00	3	-	-
4	5	17.00	1	-	-
5	10	17.00	17	7	-
6	7	14.00	10	-	-
7	10	23.00	23	16	-
8	8	19.00	5	-	-
9	9	18.00	12	-	-
10	-	25.00	25	25	25
11	-	12.00	2	0	-
12	10	20.00	10	3	-
13	2	16.00	9	5	-
14	6	11.00	3	-	-
15	-	22.00	18	14	12
16	1	12.00	10	9	-
17	-	17.00	12	7	-
18	-	17.00	17	16	-
19	-	11.00	6	5	-
20	-	19.00	8	5	-
21	8	19.00	11	11	-
22	-	15.00	4	-	-
23	-	15.00	8	8	-
24	3	6.00	2	-	-
25	-	18.00	21	20	-
26	8	10.00	4	3	-
27	12	21.00	12	5	-
28	-	7.00	6	6	-
29	-	17.00	15	9	8
30	-	23.00	15	13	-
31	-	20.00	16	9	-
32	-	15.00	15	12	5
33		7.00	2		
34	-	10.00	10	5	-
35	-	10.00	4	2	-
36	-	17.00	-	8	-
37	-	7.00	19	-	23
38	-	15.00	11	9	-
39	-	11.00	6	-	-
40	15	24.00	22	21	10
41	-	8.00	4	2	-
42	-	13.00	6		-
43	8	13.00	9	-	-
44	-	10.00	8	8	-
45	-	11.00	9	6	-
46	-	21.00	16	15	-
47	-	21.00	10	5	-
48	-	21.00	-	-	-
49	-	9.00	6	2	-
50	-	20.00	16	11	-
51	-	14.00	12	8	-
52	-	19.00	15		-
53	-	25.00	17	11	-
54	-	15.00	15	-	-
55	-	8.00	3	2	-
56	-	14.00	14	13	-
57	-	12.00	9	8	-
58	12	21.00	16	-	12
Mean	8.00	15.62	10.67	8.77	12.38
Median	8.00	15.50	10.00	8.00	11.00
Standard deviation	3.64	5.08	5.96	5.57	7.76

Table S 15: Results for the paired differences between CTSCORE PRE and CTSCORE ADM, CTSCORE ADM andCTSCORE D4, CTSCORE ADM and CTSCORE D8 and CTSCORE ADM CTSCORE D12 for the CONTROL group.

	t	DF	p–value	95 % confidence interval
CTSCORE PRE CONTROL and CTSCORE ADM CONTROL	-6.6114	23	9.572e-07	[-7.932050, -4.151283]
CTSCORE ADM CONTROL and CTSCORE D4 CONTROL	0.3905	19	0.7005	[-1.089974, 1.589974]
CTSCORE ADM CONTROL and CTSCORE D8 CONTROL	0.27641	15	0.786	[-2.51673, 3.26673]
CTSCORE ADM CONTROL and CTSCORE D12 CONTROL	0.47998	19	0.6367	[-1.848341, 2.948341]

Table S 16: Results for the paired differences between CTSCORE PRE and CTSCORE ADM, CTSCORE ADM andCTSCORE D4, CTSCORE ADM and CTSCORE D8 and CTSCORE ADM CTSCORE D12 for the NEGBAL group.

	t	DF	p–value	95 % confidence interval
CTSCORE PRE NEGBAL and CTSCORE ADM NEGBAL	-11.032	18	1.929e-09	[-10.651242, -7.243495]
CTSCORE ADM NEGBAL and CTSCORE D4 NEGBAL	7.4157	54	8.707e-10	[3.449224, 6.005321]
CTSCORE ADM NEGBAL and CTSCORE D8 NEGBAL	10.09	39	1.983e-12	[5.896615, 8.853385]
CTSCORE ADM NEGBAL and CTSCORE D12 NEGBAL	1.6615	7	0.1406	[-2.38059, 13.63059]

Figure S 7: Boxplot for CTSCORE PRE, CTSCORE ADM, CTSCORE D4, CTSCORE D8 and CTSCORE D12 for the CONTROL group.

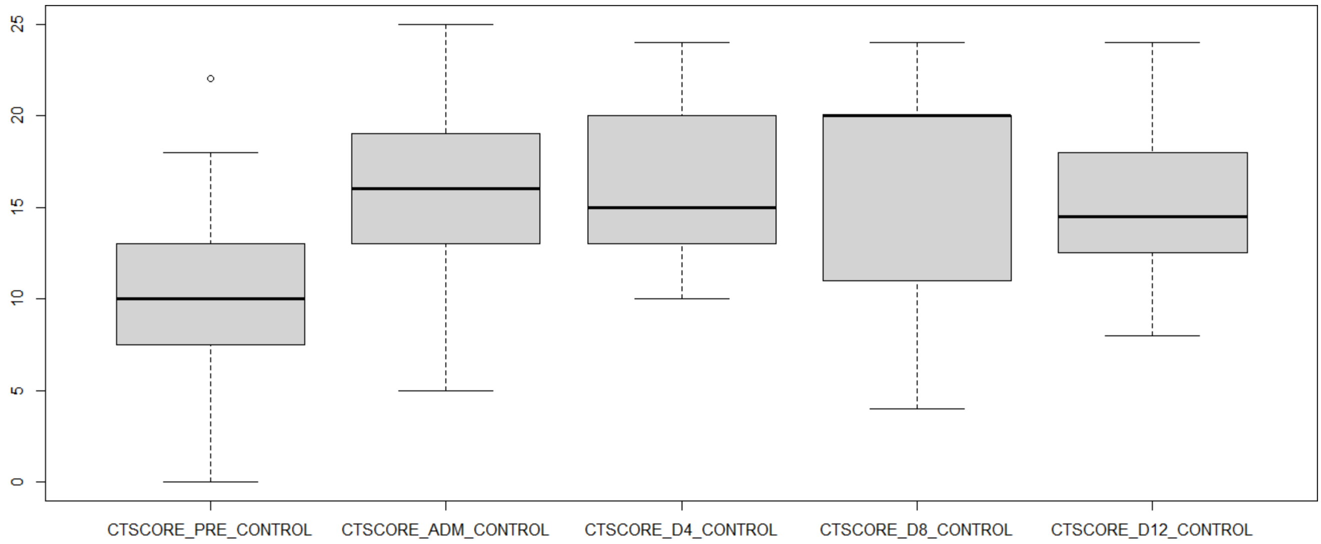

Figure S8: Boxplot for CTSCORE PRE, CTSCORE ADM, CTSCORE D4, CTSCORE D8 and CTSCORE D12 for the NEGBAL group.

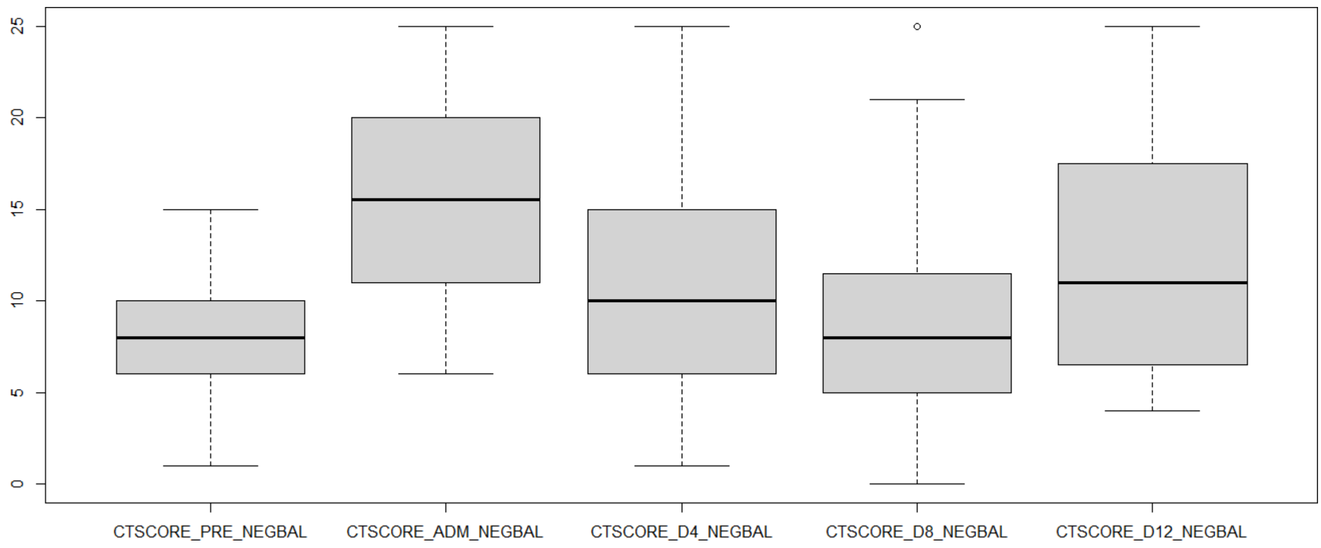

Figure S9: Boxplot for SVC PRE, SVC ADM, SVC D4, SVC D8 and SVC D12 for the CONTROL group.

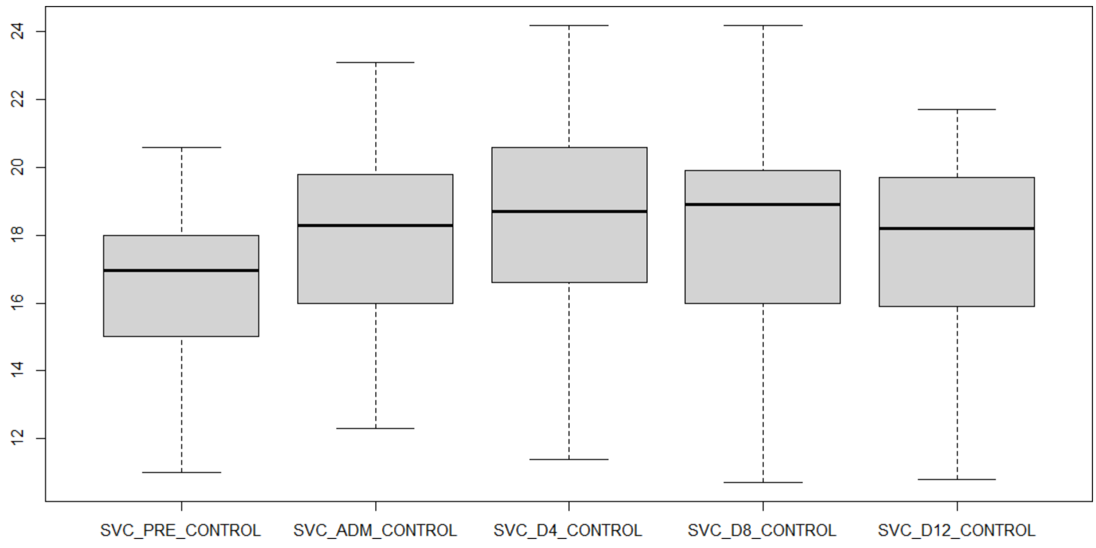

Table S17: SVC previous to the treatment and on the day of admission, the fourth, the eight and the twelfth days for the CONTROL group

	SVC_PRE_CONTROL	SVC_ADM_CONTROL	SVC_D4_CONTROL	SVC_D8_CONTROL	SVC_D12_CONTROL
1	-	12.60	-	-	10.80
2	-	19.70	16.40	-	-
3	-	19.10		19.00	19.80
4	17.80	18.40	-	-	-
5	20.60	21.40	18.70	-	-
6	-	17.80	-	-	-
7	18.10	18.30	-	-	-
8	16.00	16.00	-	-	-
9	-	-	23.50	-	-
10	-	12.30	-	-	-
11	-	15.40	11.40	-	-
12	11.30	18.10	-	-	-
13	17.00	18.60	-	-	19.00
14	12.60	14.90	-	-	21.70
15	-	13.60	-	-	15.20
16	16.00	17.40	16.10	-	14.90
17	-	-	-	13.90	-
18	17.90	16.50	-	20.60	-
19	14.80	20.20	-	21.20	19.30
20	-	19.80	20.60	-	19.60
21	-	22.00	23.90	20.00	-
22	-	15.30	-	10.70	16.10
23	-	15.90	18.00	-	17.50
24	16.90	19.00	-	-	16.70
25	-	16.00	18.70	-	-
26	-	14.70	-	-	-
27	16.10	17.50	18.00	-	-
28	15.20	19.50	19.70	19.90	20.30
29	-	18.90	16.60	16.50	17.80
30	-	17.10	-	18.10	-
31	18.10	18.70	-	-	-
32	18.30	23.10	-	-	14.30
33	-	18.10	-	15.40	-
34	-	18.00	20.20	-	-
35	11.20	21.20	-	-	-
36	15.00	15.50	-	-	-
37	11.00	17.30	-	12.10	-
38	17.10	17.90	14.80	-	-
39	-	22.80	20.80	-	-
40	17.80	18.30	16.30	-	-
41	-	-	-	-	-
42	-	22.50	24.20	24.20	-
43	-	18.50	-	-	-
44	-	18.70	-	-	-
45	18.60	19.90	19.90	-	-
46	-	20.20	-	19.10	18.60
47	-	21.00	21.60	-	-
48	-	21.50	-	18.70	15.70
49	18.60	18.70	-	16.00	-
50	15.00	21.20	-	-	19.20
51	-	21.60	19.30	18.90	-
52	-	-	-	-	-
53	-	17.20	-	-	-
54	-	16.90	-	-	19.90
55	-	15.20	-	-	16.60
56	-	16.00	-	-	-
57	17.80	19.00	18.60	19.80	20.50
58	-	14.10	-	-	-
Mean	16.20	18.13	18.92	17.89	17.68
Median	16.95	18.30	18.7	18.90	18.20
Standard deviation	2.55	2.55	3.10	3.42	2.64

.

Table S18: SVC previous to the treatment and on the day of admission, the fourth, the eight and the twelfth days for theNEGBAL group.

	SVC_PRE_NEGBAL	SVC_ADM_NEGBAL	SVC_D4_NEGBAL	SVC_D8_NEGBAL	SVC_D12_NEGBAL
1	17.1	18.9	-	16.8	-
2	-	19.1	16.4	-	15.00
3	18.4	25.2	12.4	-	-
4	12.5	16.7	11.4	-	-
5	15.1	18.3	17.4	15.9	-
6	13.9	20.1	15.5	-	-
7	22.7	25.4	26.5	17.2	-
8	8.3	20.2	13.2	-	-
9	19.2	20.9	19.4	-	-
10	-	18.3	19.4	17.4	18.2
11	-	12.3	5.5	8.1	-
12	12.8	19.5	13.6	12.5	-
13	10.7	14.5	10.8	8.1	-
14	17.1	14.4	12.2	-	-
15	-	15.9	15.1	15	15.6
16	9.5	10.4	13.9	12.6	-
17	-	17.1	10.8	14.4	-
18	-	16.1	15.5	-	-
19	-	20	9.1	15.9	-
20	-	14.2	10.1	9.5	-
21	15.1	16.4	11.8	10.26	-
22	-	19.5	9.4	-	-
23	-	15	13.9	13.5	-
24	13.7	14.9	8.3	-	-
25	-	14.6	14.1	14.1	-
26	13	12.4	10.2	10.3	-
27	17.6	22.2	15.7	13.6	-
28	-	15.6	12.8	15.6	-
29	-	22	17.8	10.3	12.6
30	-	18.9	16	16.2	-
31	-	15	11	9.5	-
32	-	13.6	13.1	9.4	6.5
33		11.8	7.2		
34	-	20	16.8	15.7	-
35	-	15.5	7	8.3	-
36	-	18.1	-	12.9	-
37	-	12.6	15.9	-	15.3
38	-	16.2	15.9	12.2	-
39	-	14	10.7	-	-
40	19.6	19.3	18.7	18.7	19.5
41	-	13.2	9.6	6.3	-
42	-	18	13.1	-	-
43	13.3	16.7	16.2	-	-
44	-	15.1	14.6	13.4	-
45	-	13,3	6.8	6.8	-
46	-	21	18.8	18.7	-
47	-	19.9	8.9	10.8	-
48	-	18	-	-	-
49	-	15.6	10.9	8.7	-
50	-	14.8	12.3	11.5	-
51	-	12.4	11.7	8.2	-
52	-	19.7	18.9	-	-
53	-	20.9	16.4	14.8	-
54	-	19.4	19	-	-
55	-	18.4	18.2	16.7	-
56	-	16.9	19.1	14.5	-
57	-	19.1	14.4	11.3	-
58	15.8	22.3	19.4	-	17.8
Mean	15.02	17.31	13.87	12.71	15.06
Median	15.10	17.10	13.9	12.9	15.45
Standard deviation	3.65	3.29	4.09	3.43	4.08

Table S19: Results for the paired differences between SVC PRE and SVC ADM, SVC ADM and SVC D4, SVC ADM andSVC D8 and SVC ADM SVC D12 for the CONTROL group.

	t	DF	*p*−value	95 % confidence interval
SVC_PRE_CONTROL and SVC_ADM_CONTROL	-4.2212	23	0.0003244	[-3.588564, -1.228103]
SVC_ADM_CONTROL and SVC_D4_CONTROL	1.1459	19	0.266	[-0.4421572, 1.5121572]
SVC_ADM_CONTROL and SVC_D8_CONTROL	1.7399	15	0.1023	[-0.2433132, 2.4058132]
SVC_ADM_CONTROL and SVC_D12_CONTROL	0.58265	19	0.567	[-1.088736, 1.928736]

Table S20: Results for the paired differences between SVC PRE and SVC ADM, SVC ADM and SVC D4, SVC ADM andSVC D8 and SVC ADM SVC D12 for the NEGBAL group.

	t	DF	*p*−value	95 % confidence interval
SVC_PRE_NEGBAL and SVC_ADM_NEGBAL	-4.3277	18	0.0004054	[-4.948927, -1.714231]
SVC_ADM_NEGBAL and SVC_D4_NEGBAL	6.9104	53	6.286e-09	[2.305374, 4.190923]
SVC_ADM_NEGBAL and SVC_D8_NEGBAL	8.6346	37	2.139e-10	[3.183007, 5.134887]
SVC_ADM_NEGBAL and SVC_D12_NEGBAL	1.9346	7	0.09429	[-0.6280079, 6.2780079]

Figure S10: Boxplot for SVC PRE, SVC ADM, SVC D4, SVC D8 and SVC D12 for the NEGBAL group.

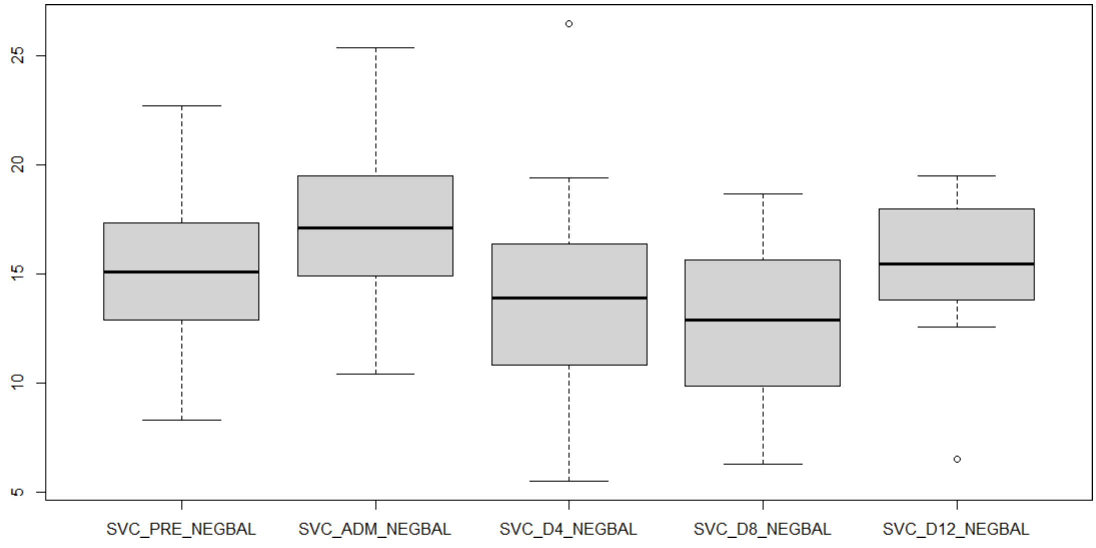

Table S21: Results for the paired differences of PAFI D4, PAFI D8 and PAFI DIS between groups.

	t	DF	$p-$value	95 % confidence interval
PAFI_D4_CONTROL and PAFI_D4_NEGBAL	-4.0626	107.41	9.254e-05	[-109.83894, -37.80063]
PAFI_D8_CONTROL and PAFI_D8_NEGBAL	-5.3513	84.538	7.352e-07	[-146.85990, -67.28795]
PAFI_DIS_CONTROL and PAFI_DIS_NEGBAL	-5.0011	111.65	2.135e-06	[-141.13147, -61.03357]

Figure S11: Boxplot for PAFI_D4_CONTROL and PAFI_D4_NEGBAL.

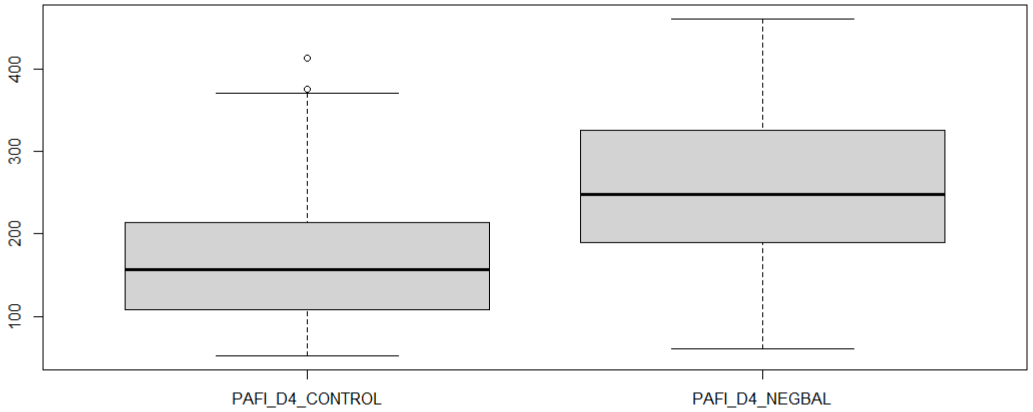

3.2 Day by day comparison

Table 21 contains the results for the paired differences between PAFI D4, PAFI D8 and PAFI DIS between the groups. Theboxplots for each of these moments are in Figures 11, 12 and 13 respectively.
Table 22 contains the results for the paired differences between HCT_D4, HCT_D8 and HCT_DIS between the groups.
The boxplots for each of these moments are in Figures 14, 15 and 16 respectively.
Table 23 contains the results for the paired differences between CREAT_D4, CREAT_D8 and CREAT_DIS between thegroups. The boxplots for each of these moments are in Figures 17, 18 and 19 respectively.
Table 24 contains the data of LEUC for the CONTROL and NEGBAL groups; the results for the paired differences canbe found in Table 25 and the corresponding boxplot in Figure 20.
Table 26 contains the results for the paired differences between CTSCORE_ADM, CTSCORE_D4, CTSCORE_D8 andCTSCORE_D12 between the groups. The boxplots for each of these moments are in Figures 21, 22, 23 and 24 respectively.
Table 27 contains the results for the paired differences between SVC_ADM, SVC_D4, SVC_D8 and SVC_D12 between thegroups. The boxplots for each of these moments are in Figures 25, 26, 27 and 28 respectively.

Tables 28 and 31 contain, respectively, the data of ACC_HYD_BAL for the CONTROL and NEGBAL groups; the results for the paired differences can be found in Table 32 and the corresponding boxplot in Figure 29. We point out the presenceof one outlier in ACC HYD BAL CONTROL. The data of ACC HYD BAL for survivors of the CONTROL group can be found in Table 29 and for nonsurvivors in Table 30.
Tables 33 and 34 contain, respectively, the data of hopsitalization days for the CONTROL and NEGBAL groups; the results for the paired differences can be found in Table 35 and the corresponding boxplot in Figures 30, 31 and 32 (for DAYS INT, ICU DAYS and DAYS MiV respectively). We point out the presence of outliers: HOSP DAYS CONTROL (1),HOSP DAYS NEGBAL (6), ICU DAYS NEGBAL (3), DAYS MiV NEGBAL (1).
Table 37 is the contingency table for the result of the treatment and the result of the χ^2 test can be found at its caption.

The $p-$value obtained was 0.001135. A mosaic plot for this can be found in Figure 33.

Table S22: Results for the paired differences of HCT D4, HCT D8 and HCT DIS between groups.

	t	DF	$p-$value	95 % confidence interval
HCT_D4_CONTROL and HCT_D4_NEGBAL	-4.0163	108.99	0.0001089	[-5.907331, -2.003513]
HCT_D8_CONTROL and HCT_D8_NEGBAL	-3.8716	81.502	0.0002171	[-6.385501, -2.050530]
HCT_ADM_NEGBAL and HCT_DIS_NEGBAL	-3.88	110.17	0.0001782	[-6.993731, -2.264890]

Figure S12: Boxplot for PAFI_D8_CONTROL and PAFI_D8_NEGBAL.

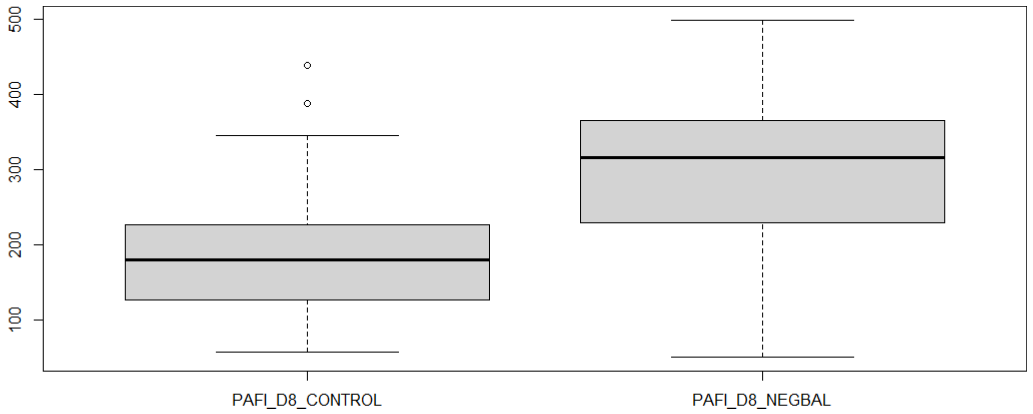

Figure S13: Boxplot for PAFI_DIS_CONTROL and PAFI_DIS_NEGBAL.

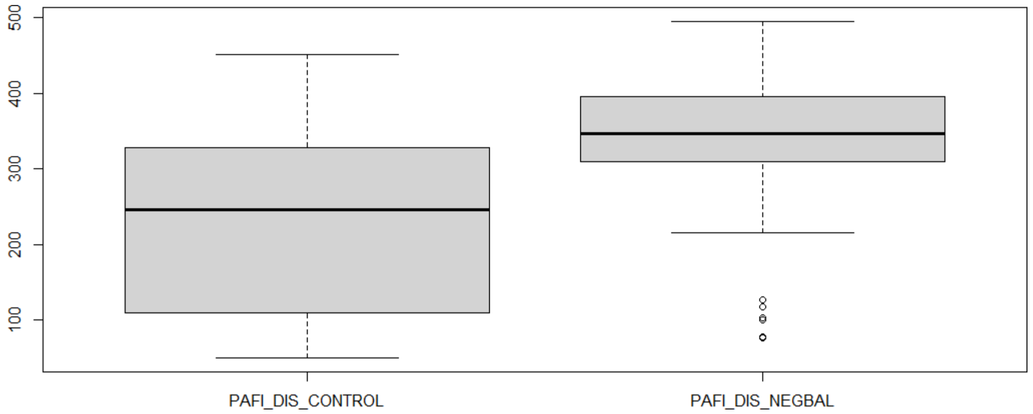

Table S23: Results for the paired differences of CREAT_D4, CREAT_D8 and CREAT_DIS between groups.

	t	DF	p–value	95 % confidence interval
CREAT_D4_CONTROL and CREAT_D4_NEGBAL	-2.1734	106.16	0.03197	[-2.9372802, -0.1348627]
CREAT_D8_CONTROL and CREAT_D8_NEGBAL	-0.14601	68.883	0.8843	[-2.879588, 2.486835]
CREAT_DIS_NEGBAL and CREAT_DIS_NEGBAL	0.88977	110.42	0.3755	[-1.845007, 4.851904]

Figure S14: Boxplot for HCT D4 CONTROL and HCT D4 NEGBAL.

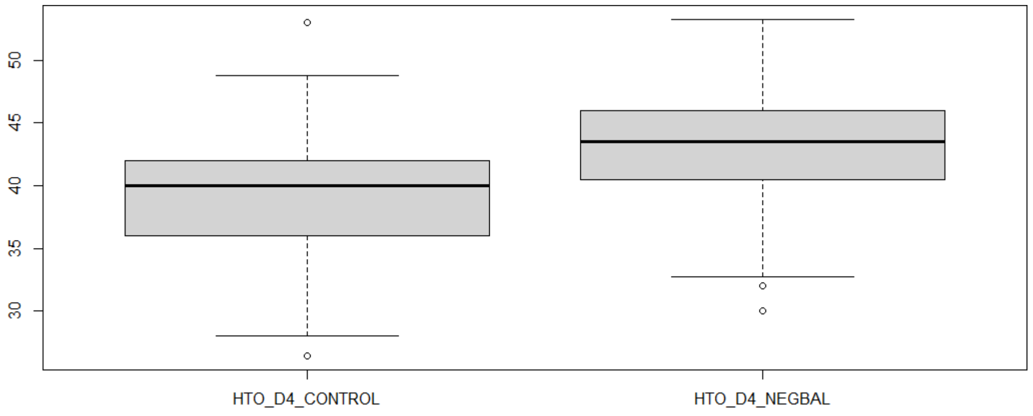

Figure S15: Boxplot for HCT D8 CONTROL and HCT D8 NEGBAL.

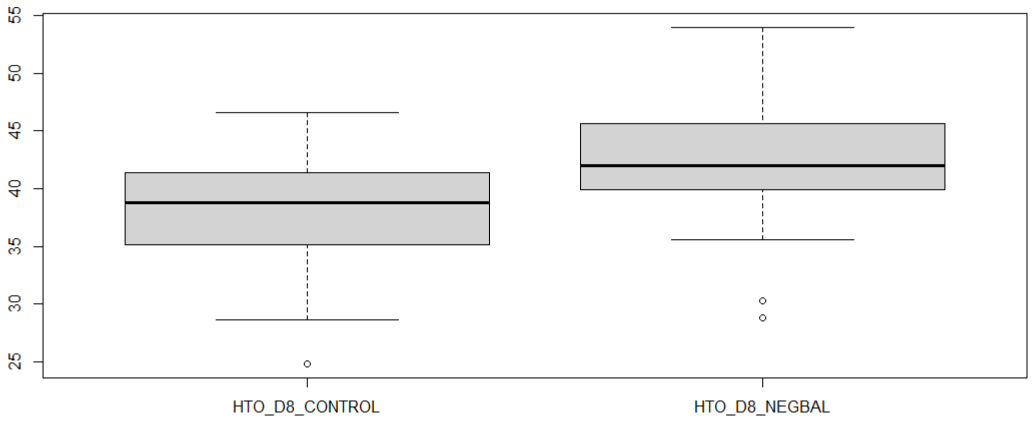

Figure S16: Boxplot for HCT DIS CONTROL and HCT DIS NEGBAL.

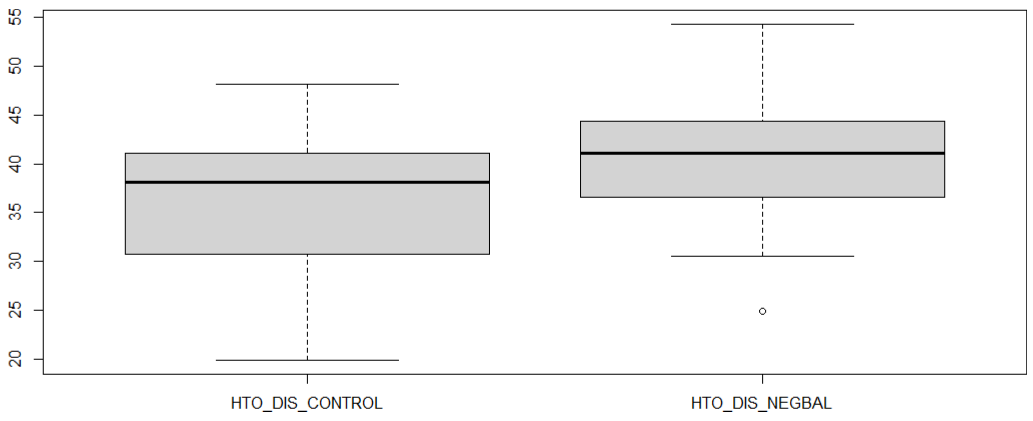

Figure S17: Boxplot for CREAT D4 CONTROL and CREAT D4 NEGBAL.

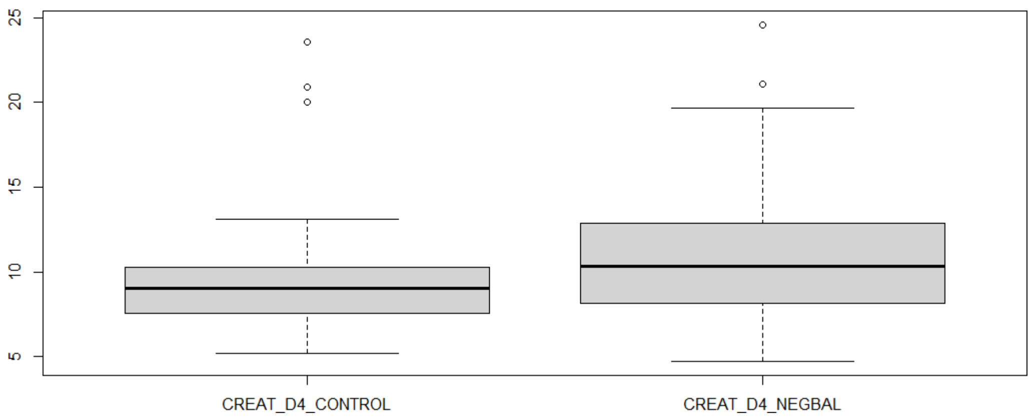

Figure S18: Boxplot for CREAT D8 CONTROL and CREAT D8 NEGBAL.

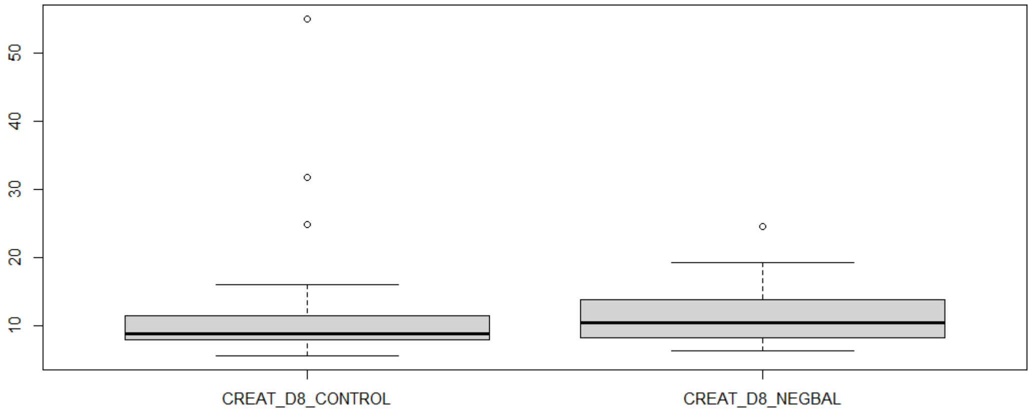

Figure S19: Boxplot for CREAT DIS CONTROL and CREAT DIS NEGBAL.

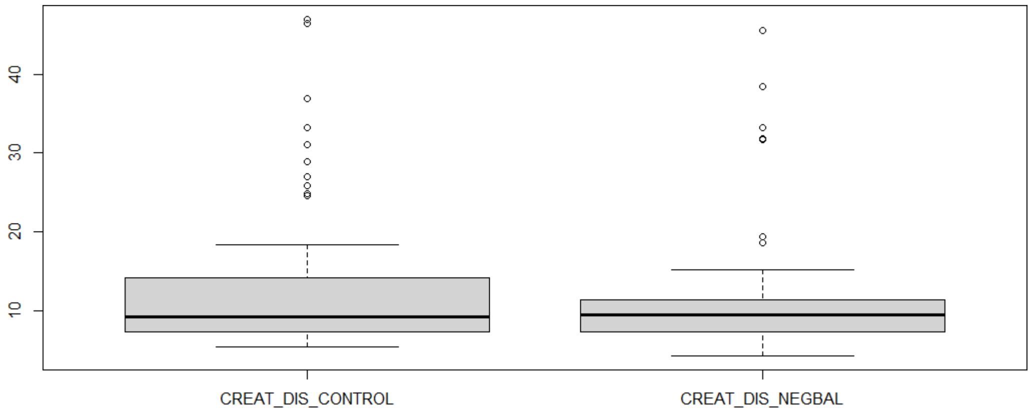

Table S24: Results LEUC ADM CONTROL and LEUC ADM NEGBAL.

	LEUC_ADM_CONTROL	LEUC_ADM_NEGBAL
1	6800	12140
2	9900	16760
3	8900	22000
4	6330	8450
5	13300	9210
6	6960	16560
7	10300	16830
8	11710	8000
9	6340	8510
10	7460	14450
11	4380	5850
12	9200	13670
13	6880	17700
14	7700	16900
15	10180	14090
16	6370	6900
17	7370	7050
18	18000	16500
19	6220	6530
20	9500	6660
21	4780	8800
22	4960	3890
23	6370	10470
24	7510	4430
25	4170	3830
26	7600	24500
27	5170	9980
28	5910	5630
29	7600	6370
30	3730	10470
31	7650	32150
32	9840	7240
33	11500	9780
34	6790	8550
35	9110	8570
36	8690	12530
37	9200	8080
38	3430	8830
39	5200	4840
40	7080	3870
41	30070	6260
42	6140	6410
43	7770	17150
44	13090	14570
45	5510	5020
46	5560	4440
47	3170	5020
48	7590	6020
49	5080	12240
50	7600	23730
51	5180	10130
52	7470	11800
53	7240	10100
54	5020	11780
55	4110	11870
56	6300	10790
57	3830	3600
58	11430	6100
Mean	7797	10597
Median	7160	9020
Standard deviation	4040.17	5796.05

Table S25: Results for the paired differences between LEUC_ADM_CONTROL and LEUC_ADM_NEGBAL.

	t	DF	p−value	95 % confidence interval
LEUC_ADM_CONTROL and LEUC_ADM_NEGBAL	-3.0173	101.81	0.003222	[-4639.2823, -958.9936]

Figure S20: Boxplot for LEUC_ADM_CONTROL and LEUC_ADM_NEGBAL.

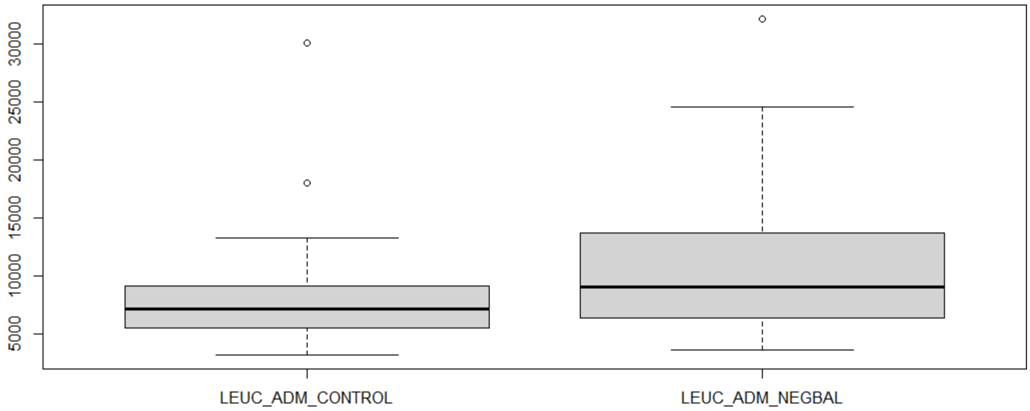

Table S26: Results for the paired differences of CTSCORE_ADM, CTSCORE_D4, CTSCORE_D8 and CTSCORE_D12 between groups.

	t	DF	p−value	95 % confidence interval
CTSCORE_ADM_CONTROL and CTSCORE_ADM_NEGBAL	0.24334	108.18	0.8082	[-1.519464, 1.944751]
CTSCORE_D4_CONTROL and CTSCORE_D4_NEGBAL	4.6922	50.014	2.13e-05	[3.319251, 8.287675]
CTSCORE_D8_CONTROL and CTSCORE_D8_NEGBAL	4.5726	29.231	8.175e-05	[4.18968, 10.96620]
CTSCORE_D12_CONTROL and CTSCORE_D12_NEGBAL	1.0991	9.0738	0.3	[-3.404303, 9.854303]

Table S27: Results for the paired differences of SVC_ADM, SVC_D4, SVC_D8 and SVC_D12 between groups.

	t	DF	p−value	95 % confidence interval
SVC_ADM_CONTROL and SVC_ADM_NEGBAL	1.4783	105.01	0.1423	[-0.2813917, 1.9303196]
SVC_D4_CONTROL and SVC_D4_NEGBAL	5.781	47.589	5.55e-07	[3.292002, 6.804275]
SVC_D8_CONTROL and SVC_D8_NEGBAL	5.2026	30.594	1.246e-05	[3.147636, 7.210373]
SVC_D12_CONTROL and SVC_D12_NEGBAL	1.6744	9.4462	0.1268	[-0.8919007, 6.1169007]

Figure S21: Boxplot for CTSCORE_ADM_CONTROL and CTSCORE_ADM_NEGBAL.

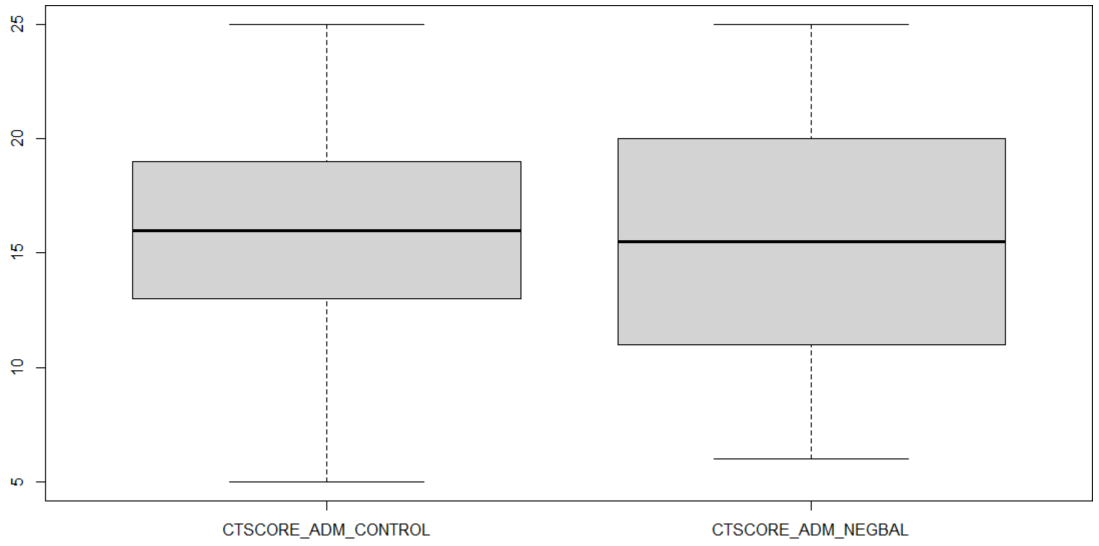

Figure S22: Boxplot for CTSCORE_D4_CONTROL and CTSCORE_D4_NEGBAL.

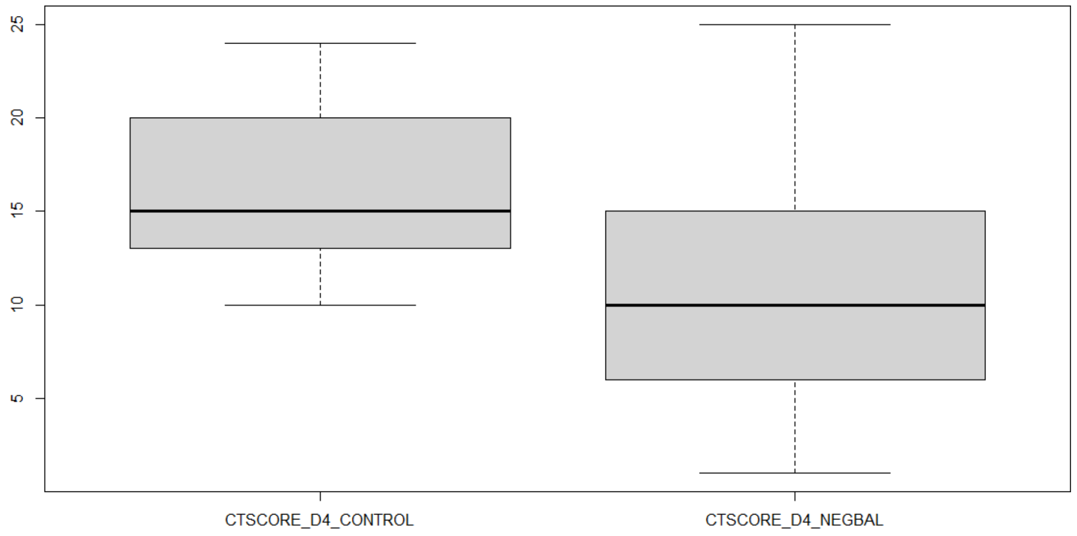

Figure S23: Boxplot for CTSCORE D8 CONTROL and CTSCORE D8 NEGBAL.

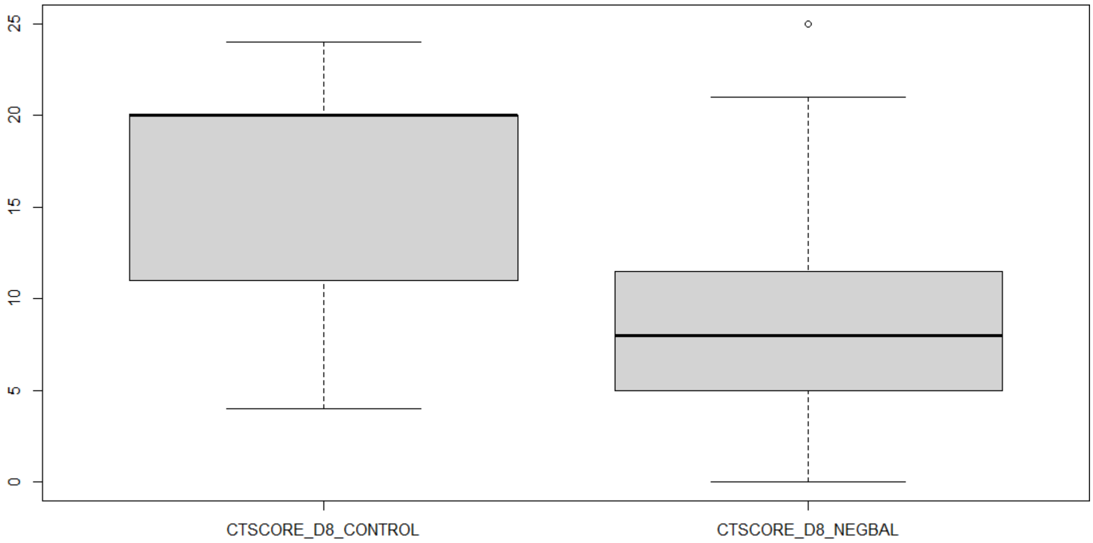

Figure S24: Boxplot for CTSCORE D12 CONTROL and CTSCORE_D12 NEGBAL.

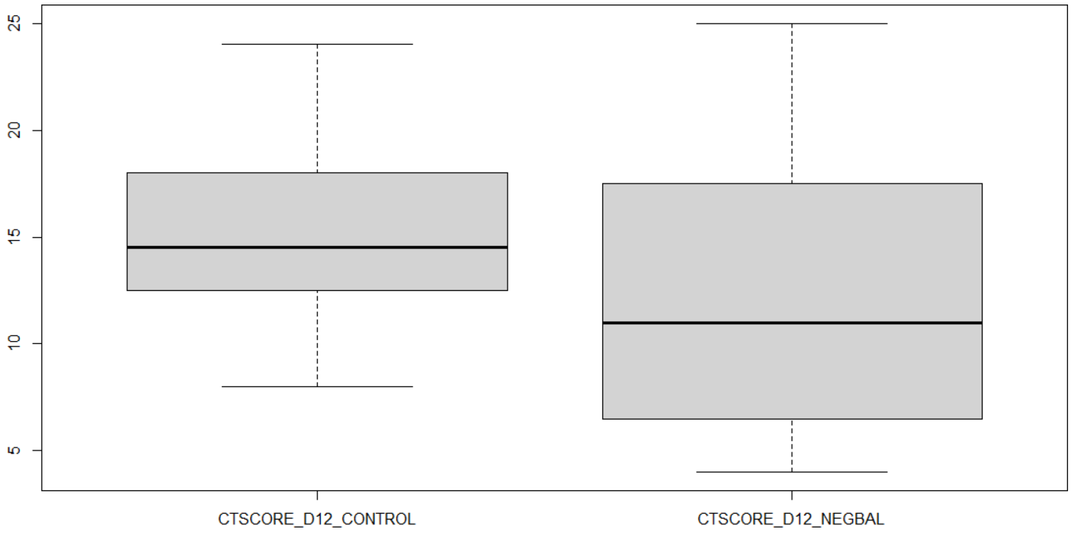

Figure S25: Boxplot for SVC ADM CONTROL and SVC ADM NEGBAL.

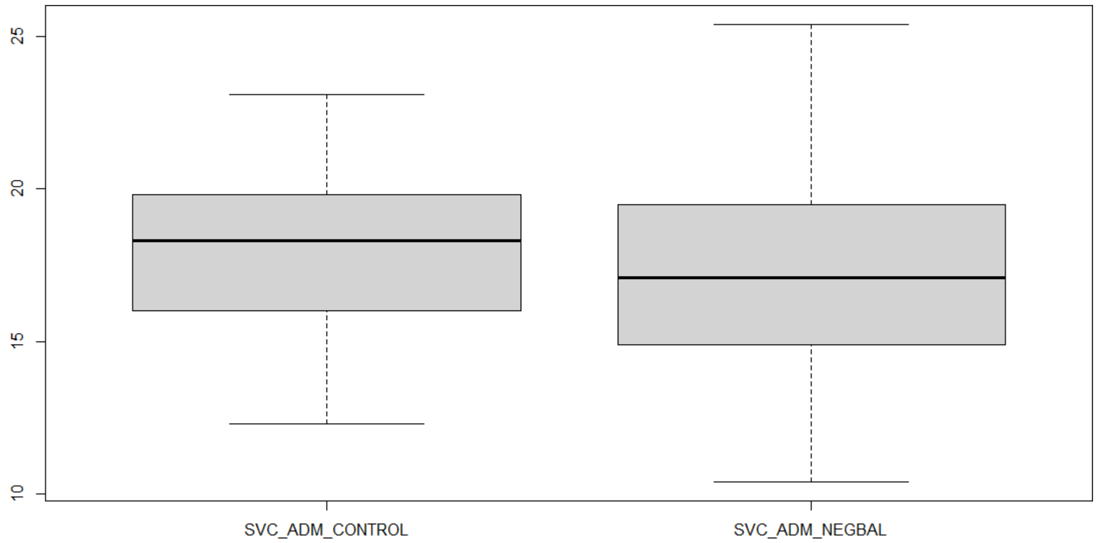

Figure S26: Boxplot for SVC D4 CONTROL and SVC D4 NEGBAL.

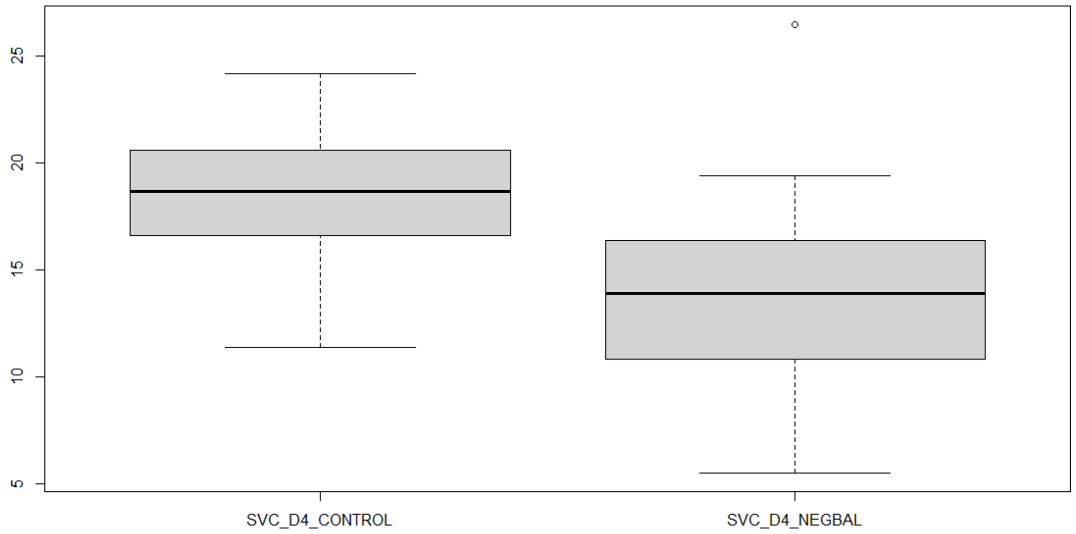

Figure S27: Boxplot for SVC D8 CONTROL and SVC D8 NEGBAL.

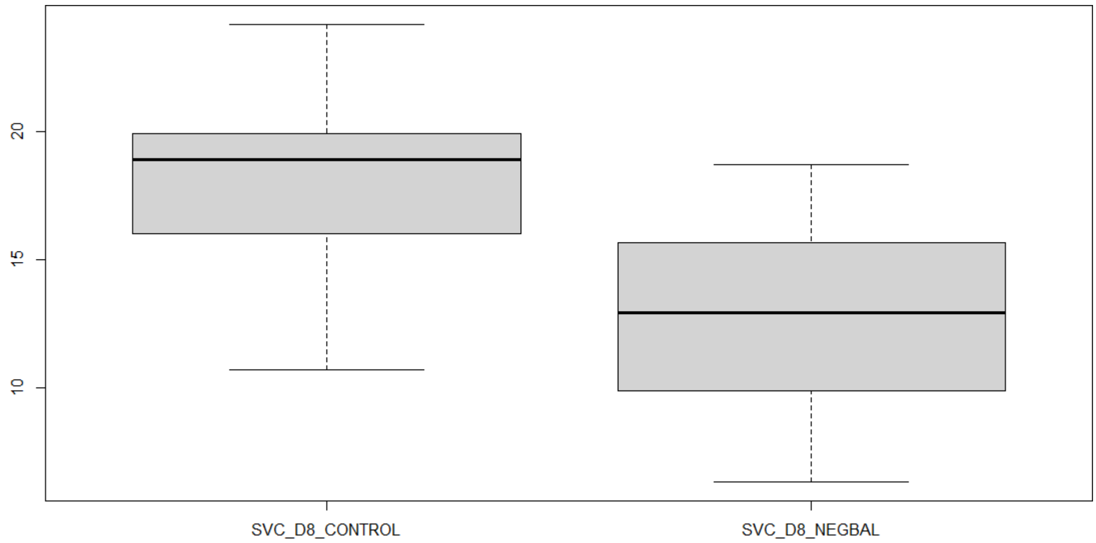

Figure S28: Boxplot for SVC D12 CONTROL and SVC D12 NEGBAL.

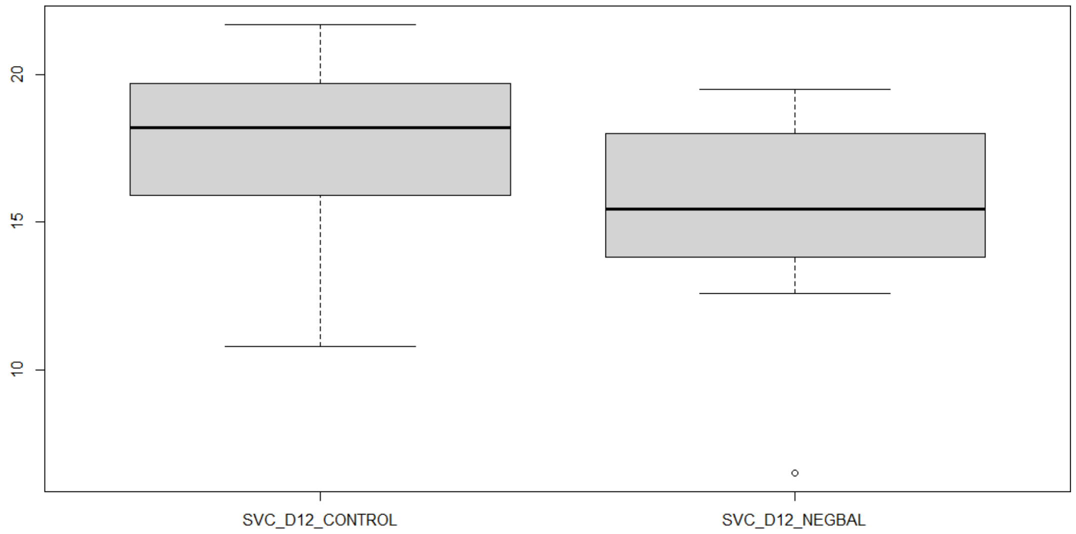

Figure S29: Boxplot for ACC HYD BAL CONTROL and ACC HYD BAL NEGBAL.

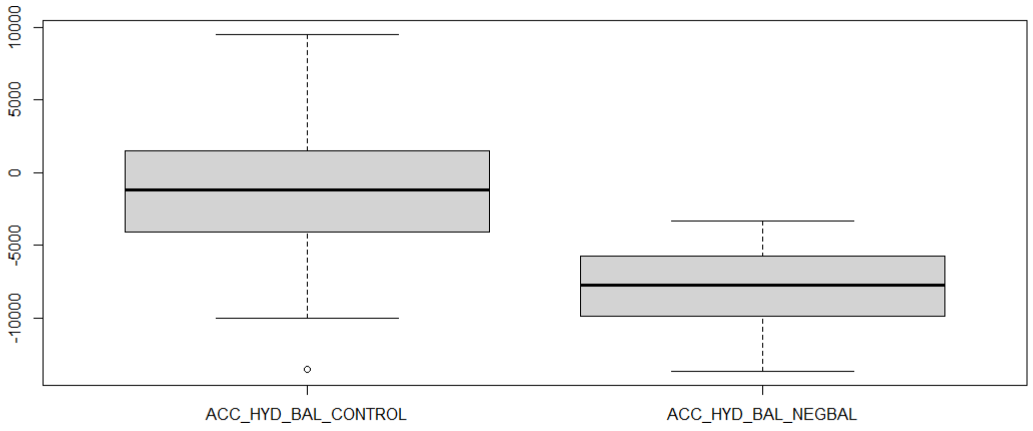

3.3 Regression

Regarding the model (1) for the CONTROL group, the p value obtained was 0.007238; the scatter plot and the regressionline can be seen in Figure 34. For the NEGBAL group the p value obtained was 0.0429; the scatter plot and the regressionline can be seen in Figure 35.

Regarding the multivariate model (2), the p−values for the variables ACC HYD BAL and VAC account for whether or not such variables are significan explaining the variation of the variable PaFiO_2. In particular, the p value obtained for the variable ACC HYD BAL was 1.43e-07, which is highly significant. As for the the variable VAC, the p values where 0.671
for the linear and 0.723 for the quadratic fit respectively, which are far from significant in both cases. A stripchart for the PaFiO_2 variable v vaccination can be found in Figure 36.

3.4 Accumulated hydric balance comparison

Figures 37, 38 and 39 contain the boxplots for ACC HYD BAL for the CONTROL group, subdivided by survivors and non survivors, the NEGBAL group, subdivided by survivors and non survivors and the whole population subdivided by survivors and non survivors respectively. The results for the paired difference tests can be found in Table 38. Table 38

Table S28: Results of Accumulated Hydric Balance of CONTROL group.

	ACC_HYD_BAL_CONTROL
1	7610
2	-6450
3	-
4	3000
5	-700
6	-
7	-1890
8	-
9	-
10	-
11	-
12	3260
13	6575
14	8900
15	-
16	170
17	1500
18	3700
19	-1363
20	5900
21	-1050
22	-5450
23	-5650
24	-2591
25	-6580
26	-
27	-
28	-4620
29	-4800
30	2151
31	-
32	-
33	-
34	-
35	-
36	-
37	-8238
38	-670
39	-4700
40	40
41	-
42	-
43	9545
44	-6670
45	-3440
46	-3810
47	-
48	-164
49	-2620
50	-
51	-13530
52	4991
53	1741
54	-
55	1196
56	900
57	-4061
58	3010
Mean	-685.0
Median	-654.2
Standard deviation	5045.215

Table S29: ACC_HYD_BAL for survivors of the CONTROL group.

	ACC_HYD_BAL_CONTROL_survivors
2	-6450
5	-700
6	-
7	-1890
8	-
9	-
10	-
11	-
16	170
19	-1363
20	5900
21	-1050
22	-5450
23	-5650
24	-2591
25	-6580
26	-
27	-
28	-4620
29	-4800
32	-
34	-
36	-
37	-8238
38	-670
40	40
44	-6670
45	-3440
46	-3810
47	-
48	-164
49	-2620
50	-
51	-13530
52	4991
54	-
56	900
Mean	-3353
Median	-2596
Standard deviation	3844.251

Table S30: ACC_HYD_BAL for nonsurvivors of the CONTROL group.

	ACC_HYD_BAL_CONTROL_nonsurvivors
1	7610
3	-
4	3000
12	3260
13	6575
14	8900
15	-
17	1500
18	3700
26	-
30	2151
31	-
33	-
35	-
39	-4700
41	-
43	9545
53	1741
55	1196
57	-4061
58	3010
Mean	1815
Median	1898
Standard deviation	4526.475

Figure S30: Boxplot for HOSP_DAYS_CONTROL and HOSP_DAYS_NEGBAL.

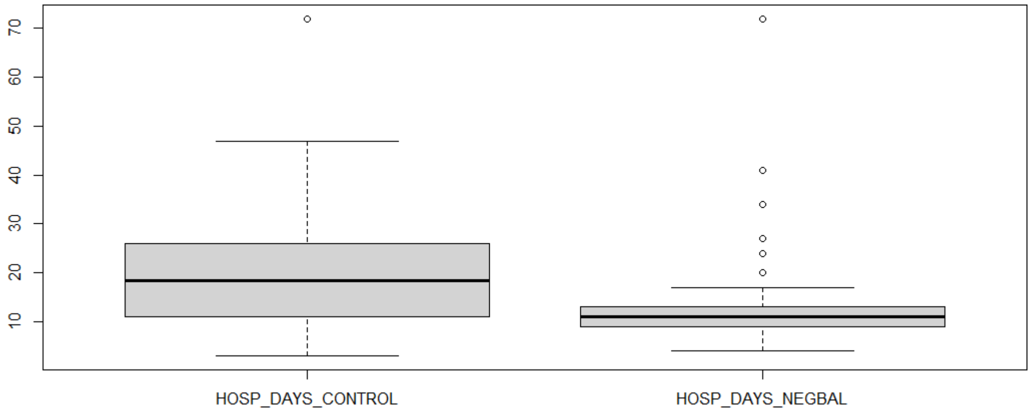

Table S31: Results of Accumulated Hydric Balance of NEGBAL group.

	ACC_HYD_BAL_NEGBAL
1	-3425
2	-11250
3	-8800
4	-6937
5	-9350
6	-10250
7	-4600
8	-10880
9	-6800
10	-7630
11	-5572
12	-9439
13	-7838
14	-7458
15	-5645
16	-5490
17	-3298
18	-9160
19	-11601
20	-9248
21	-7373
22	-6811
23	-5575
24	-5714
25	-9325
26	-6851
27	-7893
28	-5045
29	-13117
30	-11340
31	-11423
32	-7289
33	-5255
34	-4770
35	-9976
36	-7429
37	-8555
38	-9879
39	-5348
40	-12430
41	-10126
42	-7010
43	-4550
44	-12064
45	-10686
46	-10710
47	-7170
48	-5003
49	-6460
50	-8500
51	-9580
52	-8250
53	-9426
54	-8910
55	-5020
56	-6060
57	-6300
58	-13700
Mean	-8027
Median	-7734
Standard deviation	2516.09

Table S32: Results for the paired differences of ACC HYD BAL between groups.

	t	DF	p−value	95 % confidence interval
ACC HYD BAL CONTROL and ACC HYD BAL NEGBAL	9.1198	69.744	1.647e-13	[5419.504, 8453.666]

Figure S31: Boxplot for ICU_DAYS CONTROL and ICU DAYS NEGBAL.

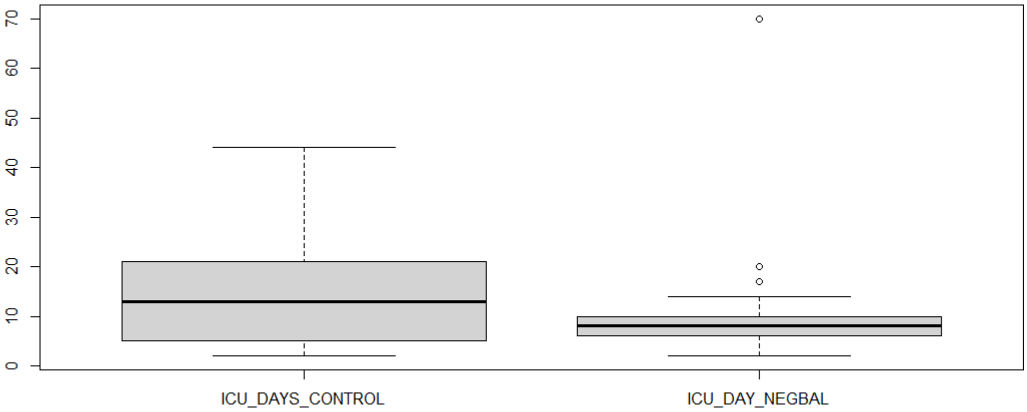

Figure S32: Boxplot for IMV DAYS CONTROL and IMV DAYS NEGBAL.

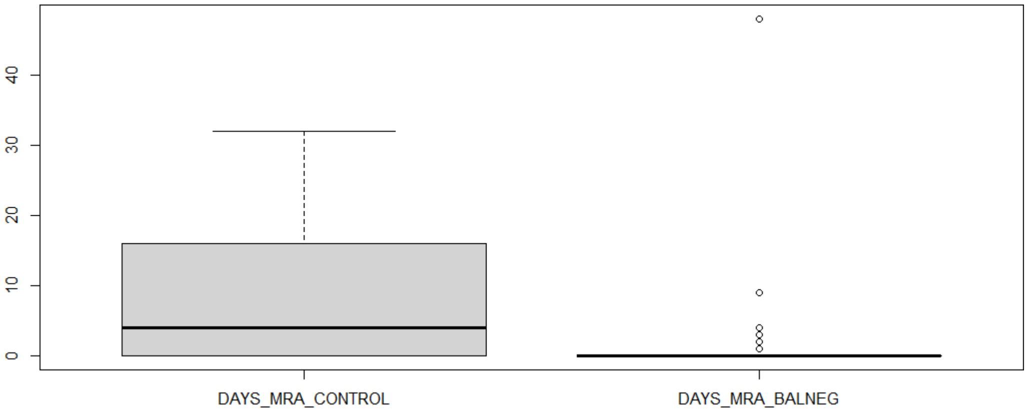

Table S33: Results of HOSP DAYS, ICU DAYS and IMV DAYS in CONTROL group.

	HOSP_DAYS CONTROL	ICU_DAYS CONTROL	DAYS MiV CONTROL
1	14	13	12
2	17	17	10
3	29	29	29
4	6	3	3
5	17	10	0
6	4	2	0
7	9	4	0
8	12	4	0
9	8	3	0
10	7	7	0
11	4	2	0
12	19	14	12
13	25	21	16
14	36	21	20
15	18	18	18
16	15	6	0
17	28	26	26
18	15	13	7
19	45	38	25
20	47	36	32
21	13	11	0
22	35	30	2
23	24	13	0
24	26	16	8
25	19	12	0
26	19	19	18
27	13	10	0
28	22	17	0
29	16	13	0
30	31	31	28
31	21	17	15
32	43	38	21
33	24	24	20
34	8	4	0
35	18	15	14
36	10	5	0
37	29	14	0
38	15	4	0
39	22	20	10
40	11	3	0
41	5	5	5
42	13	8	0
43	10	8	5
44	7	5	0
45	32	3	0
46	20	20	0
47	6	5	0
48	20	20	11
49	16	5	0
50	30	29	13
51	10	9	0
52	47	44	27
53	19	18	18
54	19	11	5
55	43	40	30
56	72	43	27
57	26	23	11
58	3	3	0
Mean	20.52	15.55	4
Median	18.50	13.00	8.58
Standard deviation	13.21	11.47	10.29

Table S34: Results of HOSP DAYS, ICU DAYS and IMV DAYS in NEGBAL group.

	HOSP_DAYS_NEGBAL	ICU_DAYS_NEGBAL	DAYS_MiV_NEGBAL
1	14	12	9
2	14	5	3
3	14	9	0
4	8	7	0
5	12	8	0
6	11	9	0
7	12	2	0
8	10	2	0
9	4	2	0
10	27	20	0
11	11	8	0
12	10	8	0
13	8	7	0
14	7	5	0
15	13	8	0
16	9	6	0
17	10	9	1
18	17	6	0
19	9	8	0
20	10	9	0
21	10	5	0
22	7	5	0
23	34	14	0
24	5	2	0
25	10	10	2
26	10	8	0
27	8	5	0
28	15	14	0
29	11	10	0
30	11	10	0
31	13	8	0
32	13	11	0
33	6	2	0
34	7	6	0
35	13	12	0
36	14	14	1
37	11	11	1
38	10	8	0
39	11	7	0
40	12	9	0
41	10	6	0
42	5	3	0
43	8	4	0
44	11	10	0
45	8	7	0
46	12	10	0
47	20	17	2
48	7	7	4
49	11	9	0
50	13	12	0
51	7	7	0
52	72	70	48
53	14	9	0
54	24	5	0
55	11	6	0
56	11	6	0
57	10	8	0
58	41	5	0
Mean	13.03	9	1.24
Median	11.00	8.00	0
Standard deviation	13.24	8.91	6.40

Table S35: Results for the paired differences of HOSP_DAYS, ICU_DAYS and IMV_DAYS between groups.

	t	DF	p−value	95 % confidence interval
HOSP_DAYS_CONTROL and HOSP_DAYS_NEGBAL	3.4249	106.7	0.0008738	[3.151546, 11.813971]
ICU_DAYS_CONTROL and ICU_DAYS_NEGBAL	3.436	107.45	0.0008405	[2.771879, 10.331569]
IMV_DAYS_CONTROL and IMV_DAYS_NEGBAL	4.6307	95.414	1.15e-05	[4.206026, 10.518112]

Table 36: Result frequencies

	Dead	Alive
CONTROL	22	36
NEGBAL	6	52

Table S37: Pearson's χ^2 test (Yates' continuity correction): χ^2= 10.593, df = 1, p−value = 0.001135

Figure 33: Mosaic plot for the result of the treatment.

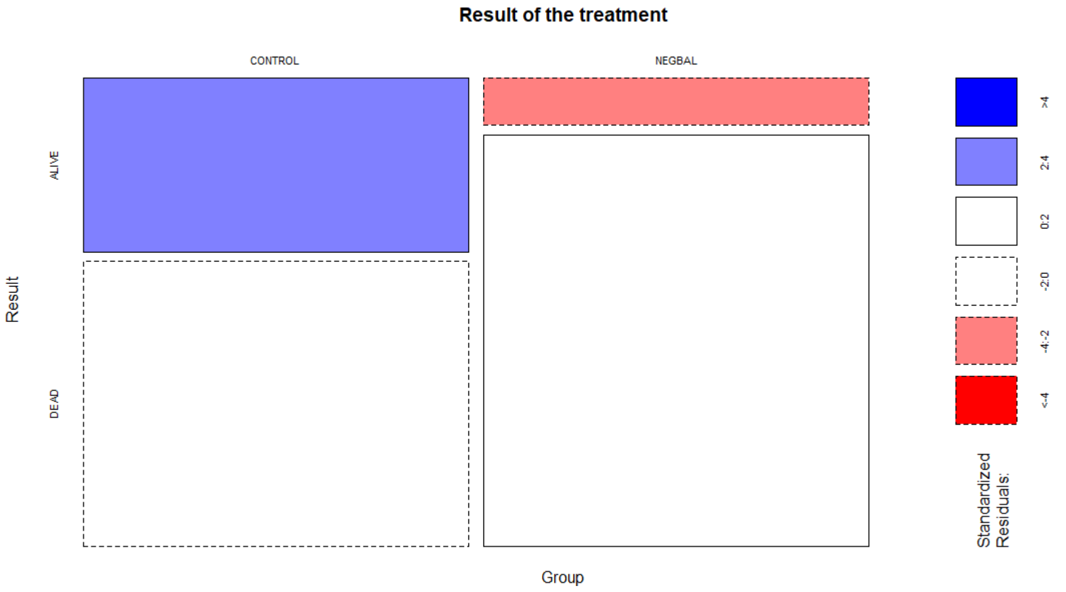

Table S38: Results for the paired differences of ACC_HYD_BAL between survivors and nonsurvivors within groups and in thewhole population.

	t	DF	p−value	95 % confidence interval
ACC_HYD_BAL_CONTROL_SUR and NONSUR	-4.2151	39.031	0.0001431	[-7648.396, -2688.247]
ACC_HYD_BAL_NEGBAL_SUR and NONSUR	-0.69792	6.4371	0.5096	[-3171.740, 1745.946]
ACC_HYD_BAL_SUR and NONSUR	-4.6943	31.437	5.002e-05	[-8035.005, -3169.679]

Figure S34: Scatter plot and regression line for ACC_HYD_BAL_CONTROL and PAFI_DIS_CONTROL, $p = 0.007238$.

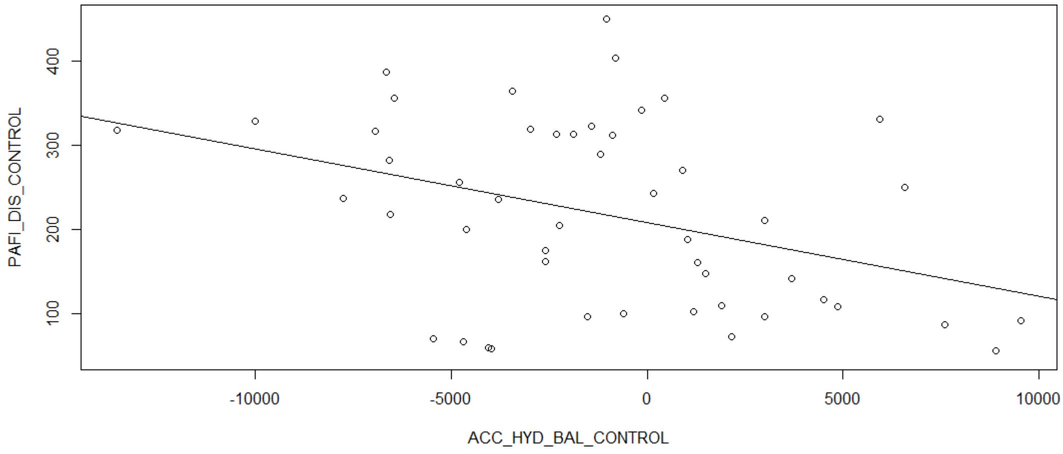

Figure S35: Scatter plot and regression line for ACC_HYD_BAL_NEGBAL and PAFI_DIS_NEGBAL , $p = 0.0429$.

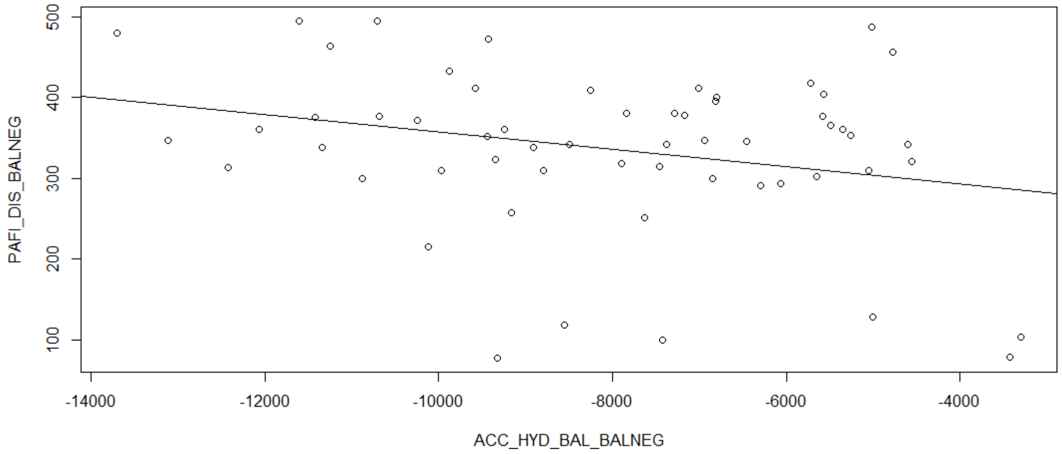

Figure S36: Stripchart for PAFI v VAC.

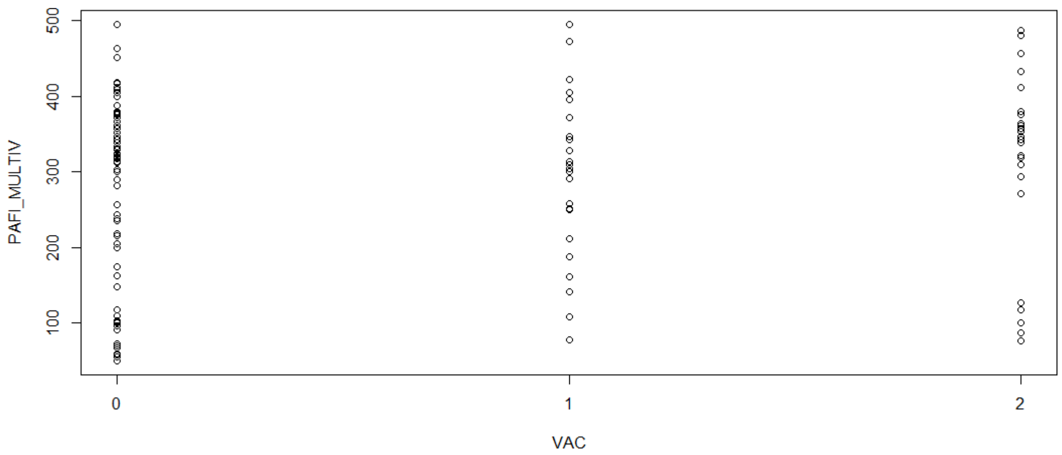

Figure S37: Boxplot for ACC HYD BAL CONTROL SUR and ACC HYD BAL CONTROL NONSUR.

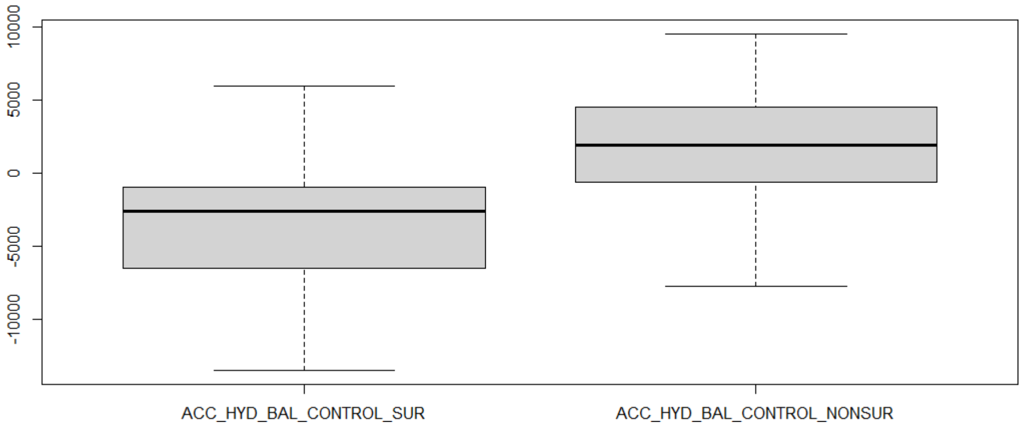

Figure S38: Boxplot for ACC HYD BAL NEGBAL SUR and ACC HYD BAL NEGBAL NONSUR.

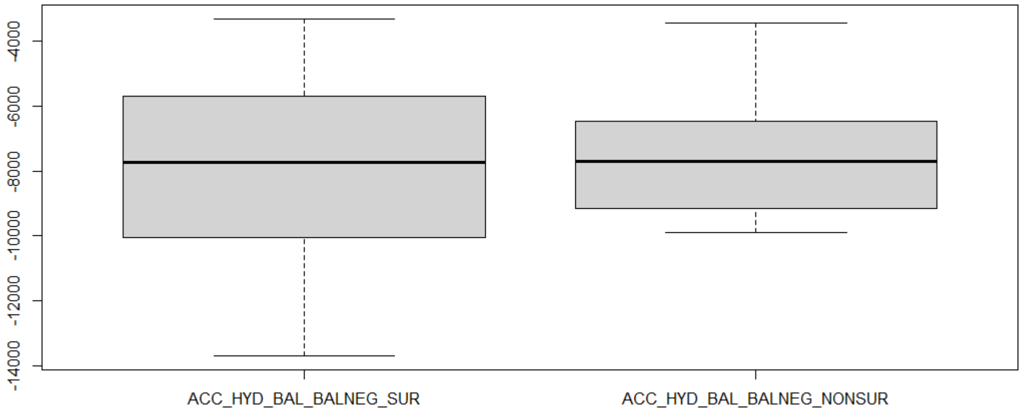

Figure S39: Boxplot for ACC HYD BAL SUR and ACC HYD BAL NONSUR.

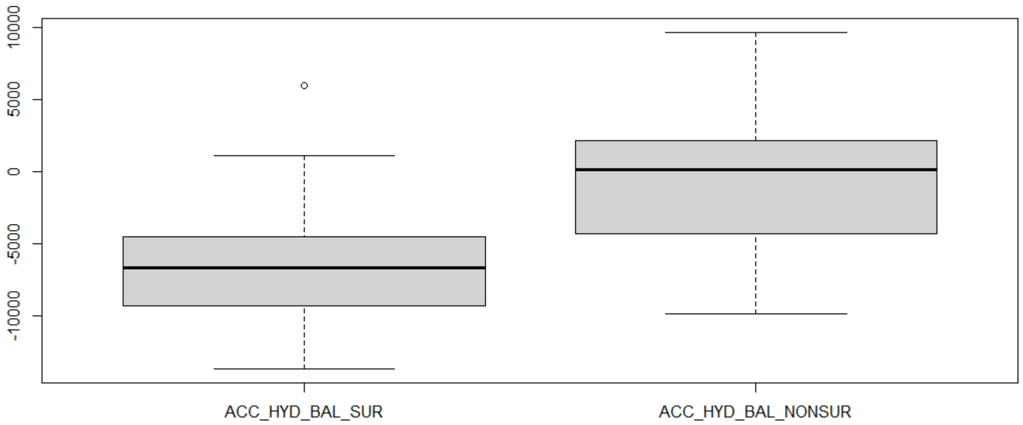

3.5 Attached Dossier

Table S39: Result IMV use

	No	Yes
CONTROL	27	31
NEGBAL	49	9

Table S40: Pearson's x^2 test (Yates' continuity correction): x^2= 16.828, df = 1, p−value = 4.093e-05

Figure S40: Boxplot for ICUfromSSD CONTROL and ICUfromSSD NEGBAL.

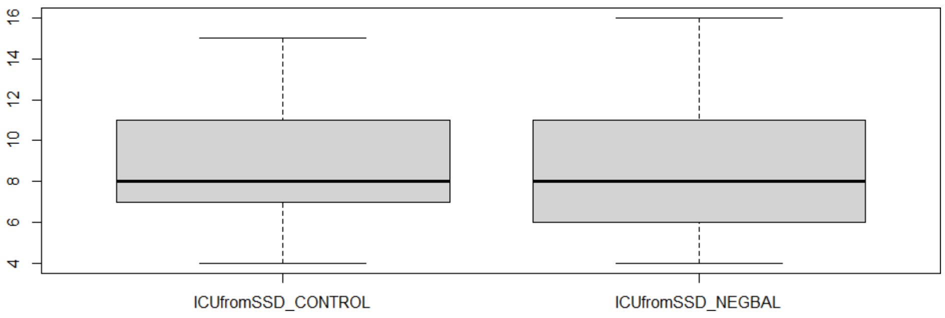

Table S39 is the contingency table for the use of Intensive Mechanical Ventilation (IMV) and the result of the x^2 test canbe found at its caption. The p value obtained was 4.093e-05.

Table S41 shows the results of the t test for the Intensive Care Unit counted from the Symptom Start Date (ICUfromSSD) comparing both groups; the corresponding boxplot can be found in Figure S40 The data is contained in Table S40.

	ICUfromSSD CONTROL	ICUfromSSD NEGBAL
1	14	5
2	7	15
3	8	10
4	15	13
5	14	11
6	10	12
7	12	15
8	7	13
9	12	13
10	6	8
11	11	10
12	8	10
13	6	9
14	10	16
15	14	14
16	11	11
17	12	14
18	9	11
19	7	7
20	7	12
21	12	10
22	8	8
23	10	5
24	7	8
25	9	5
26	7	7
27	13	7
28	5	5

29	11	11
30	7	8
31	10	6
32	7	7
33	11	5
34	8	11
35	10	6
36	8	6
37	5	4
38	6	5
39	10	9
40	10	7
41	8	11
42	8	4
43	8	9
44	6	8
45	13	8
46	8	11
47	8	7
48	7	5
49	13	4
50	7	6
51	5	8
52	5	5
53	12	4
54	9	4
55	11	12
56	10	9
57	6	12
58	4	12
Mean	8.932	8
Median	8.759	8
Standard deviation	2.73	3.28

	t	DF	p−value	95 % confidence interval
ICUfromSSD CONTROL and ICUfromSSD NEGBAL	-0.31103	110.64	0.7564	[-1.279522 0.932357]

Table S41: Results for the paired differences of ICU from SSD between groups.
